# Lentivirus-mediated IL-10-expressing Bone Marrow Mesenchymal Stem Cells promote corneal allograft survival *via* upregulating lncRNA 003946 in a rat model of corneal allograft rejection

**DOI:** 10.7150/thno.31711

**Published:** 2020-07-09

**Authors:** Xiaoxiao Lu, Yusha Ru, Chenchen Chu, Ying Lv, Yichen Gao, Zhe Jia, Yue Huang, Yan Zhang, Shaozhen Zhao

**Affiliations:** Tianjin Key Laboratory of Retinal Functions and Diseases, Tianjin International Joint Research and Development Centre of Ophthalmology and Vision Science, Eye Institute and School of Optometry, Tianjin Medical University Eye Hospital, Tianjin, 300384, China.

**Keywords:** corneal transplantation, allograft rejection, bone marrow-derived mesenchymal stem cell, Interleukin-10, long noncoding RNA

## Abstract

**Rationale:** Corneal transplantation is an effective treatment to corneal blindness. However, the immune rejection imperils corneal allograft survival. An interventional modality is urgently needed to inhibit immune rejection and promote allograft survival. In our previous study, subconjunctival injections of bone marrow-derived mesenchymal stem cells (BM-MSCs) into a rat model of corneal allograft rejection extended allograft survival for 2 d. In this study, we sought to generate IL-10-overexpressing BM-MSCs, aiming to boost the survival-promoting effects of BM-MSCs on corneal allografts and explore the molecular and cellular mechanisms underlying augmented protection.

**Methods:** A population of IL-10-overexpressing BM-MSCs (designated as IL-10-BM-MSCs) were generated by lentivirus transduction and FACS purification. The self-renewal, multi-differentiation, and immunoinhibitory capabilities of IL-10-BM-MSCs were examined by conventional assays. The IL-10-BM-MSCs were subconjunctivally injected into the model of corneal allograft rejection, and the allografts were monitored on a daily basis. The expression profiling of long noncoding RNA (lncRNA) in the allografts was revealed by RNA sequencing and verified by quantitative real-time PCR. The infiltrating immune cell type predominantly upregulating the lncRNA expression was identified by RNAscope *in situ* hybridization. The function of the upregulated lncRNA was proved by loss- and gain-of-function experiments both *in vivo* and *in vitro*.

**Results:** The IL-10-BM-MSCs possessed an enhanced immunoinhibitory capability and unabated self-renewal and multi-differentiation potentials as compared to plain BM-MSCs. The subconjunctivally injected IL-10-BM-MSCs reduced immune cell infiltration and doubled allograft survival time (20 d) as compared to IL-10 protein or plain BM-MSCs in the corneal allograft rejection model. Further, IL-10-BM-MSCs significantly upregulated lncRNA 003946 expression in CD68^+^ macrophages infiltrating corneal allografts. Silencing and overexpressing lncRNA 003946 in macrophage cultures abolished and mimicked the IL-10-BM-MSCs' suppressing effects on the macrophages' antigen presentation, respectively. In parallel, knocking down and overexpressing the lncRNA *in vivo* abrogated and simulated the survival-promoting effects of IL-10-BM-MSCs on corneal allografts, respectively.

**Conclusion:** The remarkable protective effects of IL-10-BM-MSCs support further developing them into an effective interventional modality against corneal allograft rejection. IL-10-BM-MSCs promote corneal allograft survival mainly through upregulating a novel lncRNA expression in graft-infiltrating CD68^+^ macrophages. LncRNA, for the first time, is integrated into an IL-10-BM-MSC-driven immunomodulatory axis against the immune rejection to corneal allograft.

## Introduction

Corneal transplantation is an effective therapeutics for patients with blinding corneal diseases. Although corneal transplantation has a relatively high rate of success compared with other organ transplantations, immune rejection remains a major issue of corneal allograft survival 1, and the chance of transplantation failure increases dramatically in the high-risk recipients characterized by inflamed and vascularized beds 2. Recent studies have indicated that CD4^+^ T cells and macrophages are the main culprits for immune rejection, yet the pathogenic mechanisms are not completely clear 3. The available drugs counteracting immune rejection, such as corticosteroids and immunosuppressants, have improved corneal allograft survival 4. However, local or systemic administration of these drugs causes serious side effects, including glaucoma, cataract, hypertension, and osteoporosis, thereby restricting their widespread usage 5. Therefore, an effective and safe therapeutic modality is urgently needed to promote allograft survival and improve current status of corneal transplantation.

Mesenchymal stem cells (MSCs), particularly bone marrow-derived mesenchymal stem cells (BM-MSCs), are a good candidate for such a therapeutic modality due to their anti-inflammation, anti-oxidation, multi-differentiation, and tissue repair functions 6-9. In addition, BM-MSCs migrate to sites of injury, secret immunomodulaotry cytokines, and create an amenable microenvironment through modulating natural killer cells, T and B lymphocytes, and antigen-presenting cells (APCs) 10-13. The BM-MSC therapy has been applied to corneal injury and corneal allograft rejection models 14-17. However, the protective effects on corneal transplants are inconsistent and inconclusive. In a prior study, BM-MSCs were administered to rat recipients of penetrating keratoplasty to attenuate corneal allograft rejection. However, their survival-promoting effect was limited 16. Therefore, the BM-MSCs overexpressing a gene of interest are employed in the current study to combine the advantages of cell therapy with those of gene therapy and to augment the protective effects of plain BM-MSCs 18-20.

Interleukin-10 (IL-10), a Th2 cytokine, has been shown to inhibit APC maturation and monocyte- or macrophage-dependent T cell activation 21. Further, viral vectors carrying *IL-10* cDNA have been applied in corneal transplantation and resulted in attenuated allograft rejection 22. More importantly, in our previous study, IL-10 was the only cytokine that was upregulated at the protein level in the BM-MSC-treated corneal allografts 16, indicating the crucial role of this cytokine in mediating the BM-MSC's protective effects. Therefore, we hypothesized that the genetically-engineered BM-MSCs overexpressing IL-10 may have greater effects on inhibiting immune rejection and promoting corneal allograft survival than the plain BM-MSCs. To test this hypothesis, a pure population of BM-MSCs overexpressing IL-10 (termed IL-10-BM-MSCs) were generated after lentivirus transduction and FACS purification, and this population of IL-10-BM-MSCs or plain BM-MSCs were then subconjunctivally injected into the rat model of corneal allograft rejection. The protective effects of the two types of BM-MSCs were compared at the molecular, cellular, and systemic levels.

As for the mechanism underlying the protection granted by IL-10-BM-MSCs, long noncoding RNA (lncRNA) was selected as the molecular candidate. LncRNA is a type of transcript longer than 200 nucleotides but with no protein-coding ability 23. LncRNA interacts with chromatin, other RNA species, and proteins as a signal, guide, decoy, or scaffold 23, 24, playing regulatory roles in physiological processes, including chromatin modifications, transcription, and translation 25, 26; as well as in pathogenesis and prognosis of diseases, such as cancer and diabetes 27, 28. Interestingly, accumulating evidence has demonstrated that lncRNAs regulate function and homeostasis of cell populations during immune responses 29. For example, lncRNA-Cox2 facilitates activation of late inflammatory gene transcription in macrophages challenged by bacterial infection 30. Moreover, lncRNA-GAS5 is significantly upregulated and promotes M1 macrophage polarization in peripheral blood of patients with childhood pneumonia 31. In particular, in an allogeneic mouse heart transplantation model, lncRNA-A930015D03Rik and mouselincRNA1055 were differentially expressed in grafts and infiltrating lymphocytes, thereby promoting differentiation and cytokine secretion of Th1 cells. These results implicate that differential expression of lncRNA in a particular type of immune cells may be a crucial regulator during immune responses, such as an allograft rejection 32. Therefore, we extended our hypothesis that IL-10-BM-MSCs may generate greater suppressing effects on immune rejection and promote corneal allograft survival by inducing the differential lncRNA expression in infiltrating immune cells in the rat corneal allograft rejection model. To test the extended hypothesis, a high-throughput RNA sequencing was preformed to screen the differentially expressed lncRNA, and the RNAscope *in situ* hybridization was employed to identify the immune cells harboring the differentially expressed lncRNA in the corneal allograft rejection model. Then the molecular and cellular targets mediating IL-10-BM-MSCs' protective effects were confirmed by knockdown and overexpression experiments. LncRNA, for the first time, is linked to the molecular signaling downstream IL-10-BM-MSCs against corneal allograft rejection.

## Results

### Subconjunctival delivery of BM-MSCs suppressed corneal allograft rejection and upregulated IL-10 expression

**Figure 1:**
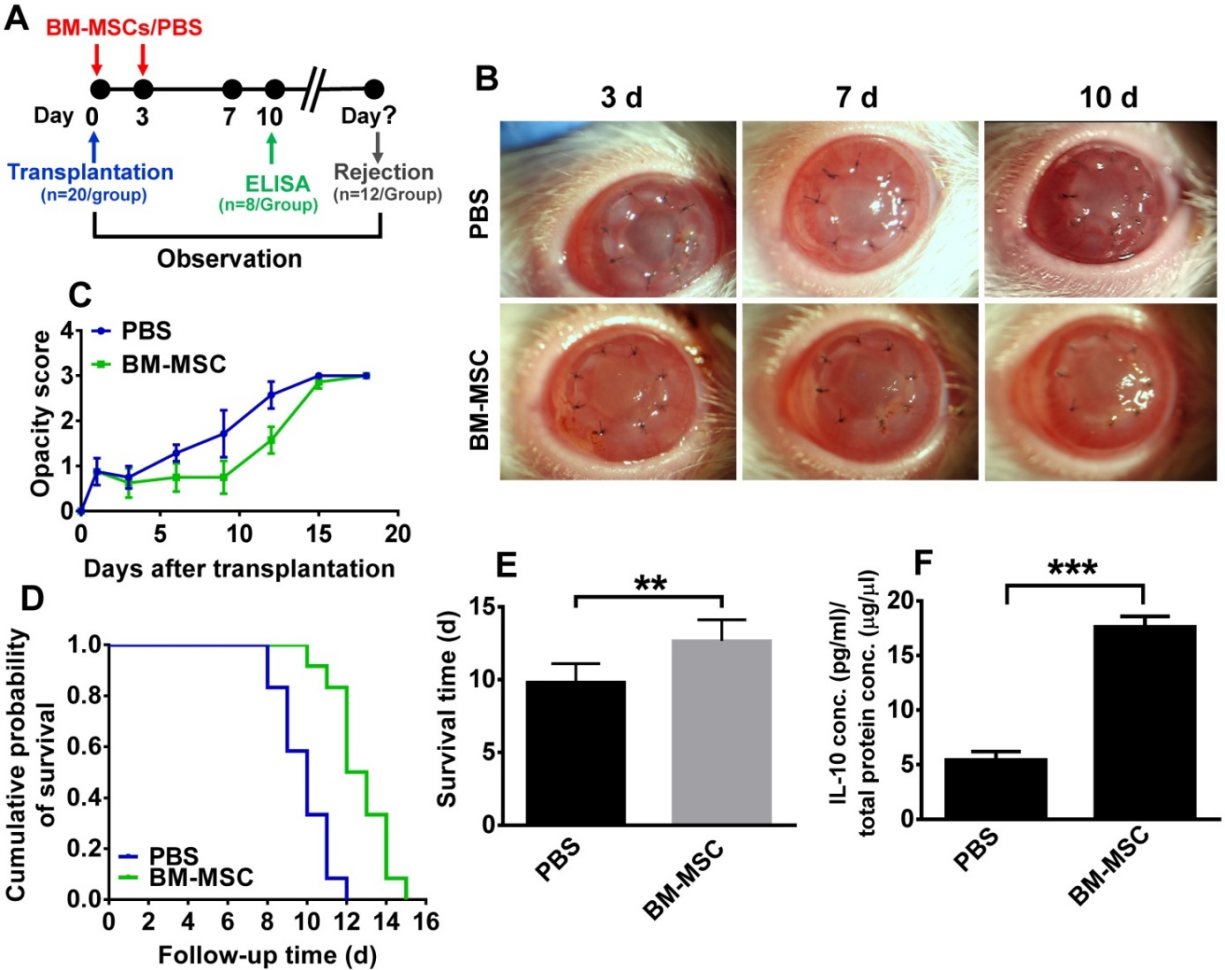
** Subconjunctival injections of BM-MSCs suppressed allograft rejection and upregulated IL-10 expression.** (**A**) Schema of BM-MSC administration in a rat model of corneal allograft rejection. (**B**) Representative pictures of corneal allografts at 3, 7, and 10 d following penetrating keratoplasty. (**C**) The opacity scores of corneal allografts following transplantation (n = 12 / group). (**D**) Kaplan-Meier survival curves of corneal allografts in experimental groups (n = 12 / group). (**E**) Comparison of the mean survival time of corneal allografts in experimental groups (n = 12 / group). (**F**) The concentrations (pg/ml) of IL-10 in the corneal allografts and recipient beds were measured at 10 d post transplantation by an ELISA and normalized to the total protein concentrations (µg/µl) (n = 8 / group). ** p < 0.01, *** p < 0.001.

To investigate whether BM-MSCs could promote allograft survival in a corneal transplantation rejection model, BM-MSCs were subconjunctivally injected immediately and at 3 d after transplantation, then the opacity, edema, and neovascularization of the allografts were monitored and scored everyday under a slit-lamp biomicroscope (Figure 1A). At each time point, the corneal allografts in phosphate-buffered saline (PBS) group were edematous and turbid in the center, with neovessels growing from the periphery. By contrast, the allografts in BM-MSC-treated group were smooth and transparent with less edema and no neovessel (Figure 1B). For corneal allograft opacity, the scores of both groups were similar during the first 3 d post transplantation, after which the score of allograft opacity in BM-MSC group was reduced, albeit not statistically significant, as compared to PBS group (Figure 1C, all p > 0.05, PBS group vs BM-MSC group). Moreover, Kaplan-Meier survival curve and the bar graph showed that the allograft survival time was 12.67 ± 1.44 d in BM-MSC group and 9.83 ± 1.27 d in PBS group (Figure 1D and E). These results suggested a limited (only 2.84 d) but significant extension in the corneal allograft survival caused by local BM-MSC administration (Figure 1E, p = 0.001, PBS group vs BM-MSC group).

To ascertain the cytokine microenvironment around the BM-MSC-treated corneal allografts, the expression profiling of Th1 and Th2 cytokines, including IFN-γ, IL-2, IL-4, and IL-10 in the corneal grafts and recipient beds was examined in a previous study 16. It turned out that only IL-10 protein levels were significantly elevated in the corneas of the MSC-treated group as compared to those in PBS group. This result was confirmed in the current study (Figure 1F, p < 0.05, PBS vs BM-MSC), suggesting that IL-10 in the local microenvironment may be crucial for BM-MSCs to inhibit rejection and promote survival of corneal allografts.

### Generation of lentivirus-mediated IL-10-expressing BM-MSCs

Since two-day extension of the allograft survival was rather limited, we next sought to enhance the protective effects of BM-MSCs. We used lentivirus to transduce BM-MSCs and generate a pure population of BM-MSCs that overexpress IL-10, thereby augmenting the immune inhibitory and protective effects of the BM-MSCs.

The rat IL-10 encoding cDNA was amplified, whose relative concentration was compared to the linearized lenti-vector (Supplementary Figure 1A). Following ligation, the positive clones were screened by regular PCR (Supplementary Figure 1B). After transducing the BM-MSCs with the lentivirus carrying *IL-10* cDNA, flow cytometry showed a transduction efficiency of nearly 50% (Supplementary Figure 1C). The transduced BM-MSCs were then sorted according to RFP expression. The sorted BM-MSCs, designated as IL-10-BM-MSCs, reached the purity of approximately 90% as revealed by flow cytometry (Supplementary Figure 1C). The presence *WPRE* fragment implicated integration of the lentiviral vector sequence into the IL-10-BM-MSC's genome (Supplementary Figure 1D). The red fluorescence of RFP was localized in the cytoplasm of the BM-MSCs transduced with the virus. The morphology of BM-MSCs before and after sorting was similar (Supplementary Figure 1E).

### Characterization of IL-10-BM-MSCs

**Figure 2:**
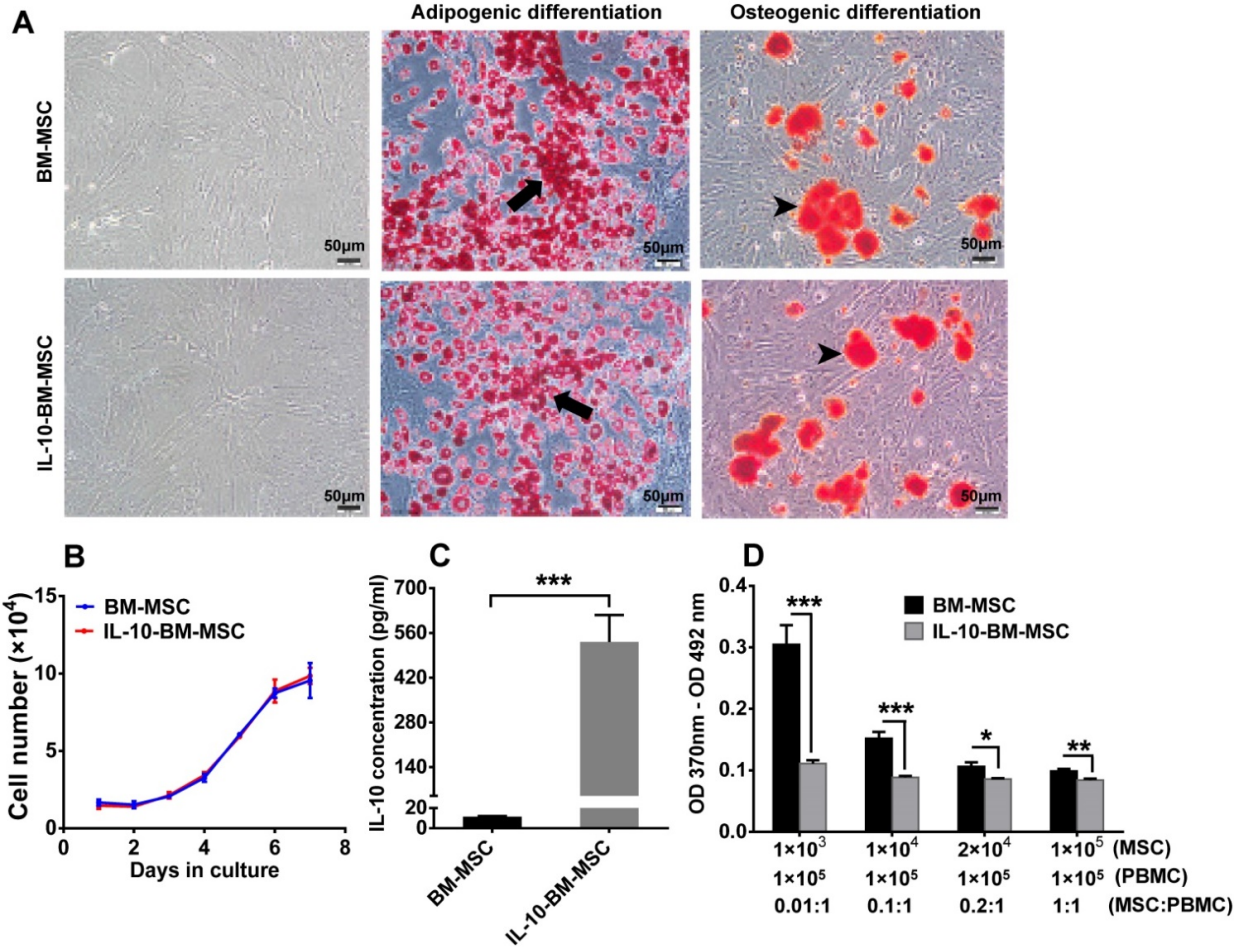
** Characterization of IL-10-BM-MSCs.** (**A**) The morphology of BM-MSCs and IL-10-BM-MSCs at passage 3 under a light microscope was shown. Both BM-MSCs and the genetically-engineered IL-10-overexpressing counterparts have the adipogenic and osteogenic potentials as indicated by Oil Red O and Alizarin Red S staining, respectively. The black arrows indicate intracellular lipid droplets (red). The black arrowheads indicate extracellular calcium deposits (orange red). Scale bar = 50 µm. (**B**) The growth curve of BM-MSCs and IL-10-BM-MSCs (n = 3 / group). (**C**) The concentration of IL-10 secreted by IL-10-BM-MSCs at the 3^rd^ passage was compared to that of plain BM-MSCs at the same passage (n = 4 / group). (**D**) The immunoinhibitory activities of BM-MSCs and IL-10-BM-MSCs were compared by coculturing different proportions of the MSCs with activated PBMCs. Then the proliferation of the PBMCs was determined by a BrdU assay (n= 6 / group). * p < 0.05, ** p* <* 0.01, *** p < 0.001.

The majority of the transduced BM-MSCs displayed a long, spindle-like shape under a light microscope, which is similar to the untransduced BM-MSCs, indicating that lentivirus transduction and FACS sorting did not change the morphology of BM-MSCs (Figure 2A).

The potential of multi-lineage differentiation was examined in the BM-MSCs and IL-10-BM-MSCs both of the 3^rd^ passage. Following a 20-d-induction for adipogenesis, lipid droplets were discernible in both batches of MSCs, and the number of the cells positive for Oil Red O staining was similar between the two cell batches. In addition, the area and intensity of alizarin red staining for extracellular calcium deposits after osteogenic induction were comparable between BM-MSCs and IL-10-BM-MSCs (Figure 2A). These results suggested an uncompromised potential of multi-lineage differentiation of BM-MSCs at 3^rd^ passage following virus transduction and FACS purification.

Moreover, BM-MSCs and IL-10-BM-MSCs demonstrated almost identical growth curves, which exhibited gradual increments from Day 0 to 4 after seeding. The cell number showed a close to linear increase from Day 4 to 6; on Day 7, the cell proliferation speed was slightly reduced (Figure 2B). This result suggested the generation of IL-10-overexpressing BM-MSCs did not affect the self-renewal ability of the BM-MSCs.

Importantly, the concentration of IL-10 in the supernatant of the 3^rd^ passage IL-10-BM-MSCs was 531.96 ± 84.31 pg / ml; it was merely 11.37 ± 0.78 pg / ml in the supernatant of plain BM-MSCs at the same passage. The IL-10 secretion was boosted 46.79 fold after lentivirus-mediated transduction and purification (Figure 2C, p < 0.001, BM-MSCs vs IL-10-BM-MSCs).

To determine the effect of IL-10-BM-MSCs on T cell proliferation, the irradiated MSCs and activated T cells were cocultured. The results of Bromodeoxyuridine (BrdU) assay showed that the BM-MSCs inhibited proliferation of activated T cells in a dose-dependent manner, with the highest proportion of BM-MSCs exerting the greatest inhibition (Figure 2D). In contrast, IL-10-BM-MSCs at four different proportions generated similar inhibitory effects on the proliferative T cells. However, IL-10-BM-MSCs at each proportion exerted significantly greater inhibition than the plain BM-MSC counterparts (Figure 2D) (BM-MSCs vs IL-10-BM-MSCs, p < 0.001, for 1 × 10^3^ and 1 × 10^4^ MSCs; p < 0.05, for 2 × 10^4^ MSCs; p < 0.01, for 1 × 10^5^ MSCs). These results implicate that the immunoinhibiotry capability of IL-10-BM-MSCs was augmented more than 100 fold as compared to plain BM-MSCs and the capability is saturable.

In general, the characterization of IL-10-BM-MSCs suggests that following virus transduction and purification, the BM-MSCs maintain self-renewal and multi-differentiation potentials, and dramatically enhance the immune inhibitory capability.

### Subconjunctival administration of IL-10-BM-MSCs substantially prolonged corneal allograft survival

**Figure 3:**
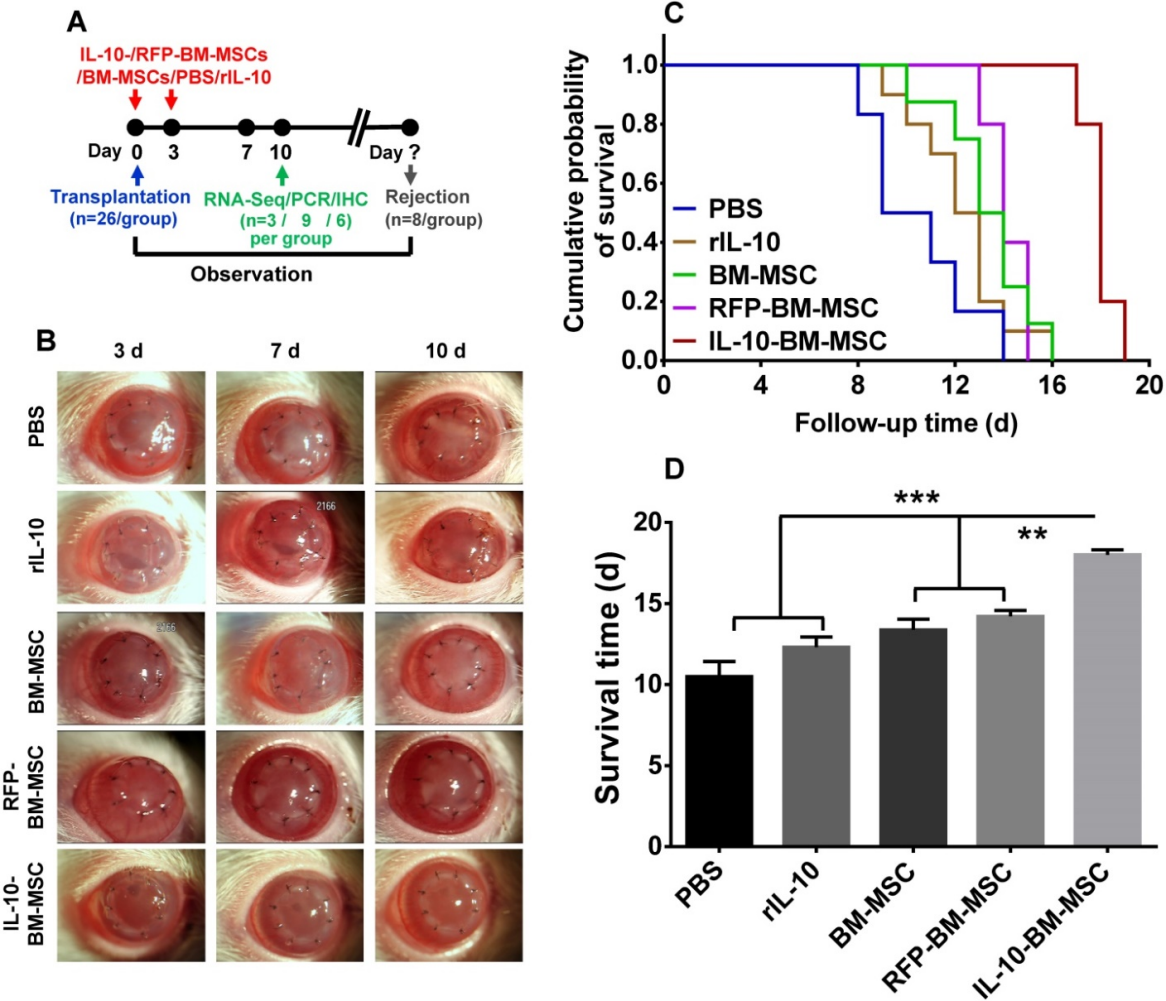
** Subconjunctival injections of IL-10-BM-MSCs substantially prolonged survival of corneal allografts.** (**A**) Corneal grafts harvested from Wistar donors were transplanted into Lewis host beds. BM-MSCs, rIL-10, or IL-10-BM-MSCs were subconjunctivally injected immediately (day 0) and at 3 d after transplantation (day 3). (**B**) Representative images of corneal allografts at day 3, 7, and 10 were shown. (**C**) Kaplan-Meier survival curves depicted corneal allograft survival in experimental groups (n = 8 / group). (**D**) The mean corneal allograft survival time was compared among the experimental groups (n= 8 / group). **p < 0.01, ***p < 0.001.

To explore the effect of IL-10-BM-MSCs on allograft survival, the corneal allograft transplantation model was established and the recombinant rat IL-10 (rIL-10), or engineered or plain BM-MSCs were subconjunctivally injected (Figure 3A). The allografts of PBS group were opaque and with severe edema. By contrast, the transplanted corneas of IL-10-BM-MSC-treated group were clear and with no edema; whereas the allografts in the groups treated with rIL-10, plain BM-MSC, or empty vector-transduced BM-MSCs (termed as RFP-BM-MSCs) were similar, being mildly turbid and edematous (Figure 3B). Consistently, Kaplan-Meier survival curve and bar graph showed that the grafts in all the treatment groups survived longer than the PBS group (Figure 3C and D, p = 0.303, PBS vs rIL-10; p < 0.05, PBS vs BM-MSC; p < 0.05 PBS vs RFP-BM-MSC); the grafts' mean survival time in the rIL-10-treated group was similar to that in the BM-MSC- and RFP-BM-MSC-treated groups (Figure 3C and D, p = 0.703, rIL-10 vs BM-MSC; p = 0.307, rIL-10 vs RFP-BM-MSC), suggesting the similar efficacies between rIL-10 cytokine and BM-MSCs in promoting graft survival and the unaffected protective functions of BM-MSCs following viral transduction and purification. It also bears noting that local administration of IL-10-BM-MSCs extended the corneal allograft survival time almost 2 fold, from 10 d to 20 d following transplantation (Figure 3C and D, p < 0.001, PBS vs IL-10-BM-MSC). The survival time of the allografts in the IL-10-BM-MSC group was significantly longer than rIL-10, BM-MSC, and RFP-BM-MSC groups (Figure 3C and D, p < 0.001, rIL-10 vs IL-10-BM-MSC; both p < 0.01, for BM-MSC vs IL-10-BM-MSC and RFP-BM-MSC vs IL-10-BM-MSC). These results indicate a promoting interaction between rIL-10 and BM-MSCs, the culmination of which is reflected at the remarkable protective effects of the IL-10-BM-MSCs on the corneal allografts.

### Administration of IL-10-BM-MSCs dramatically inhibited immune cell infiltration

**Figure 4:**
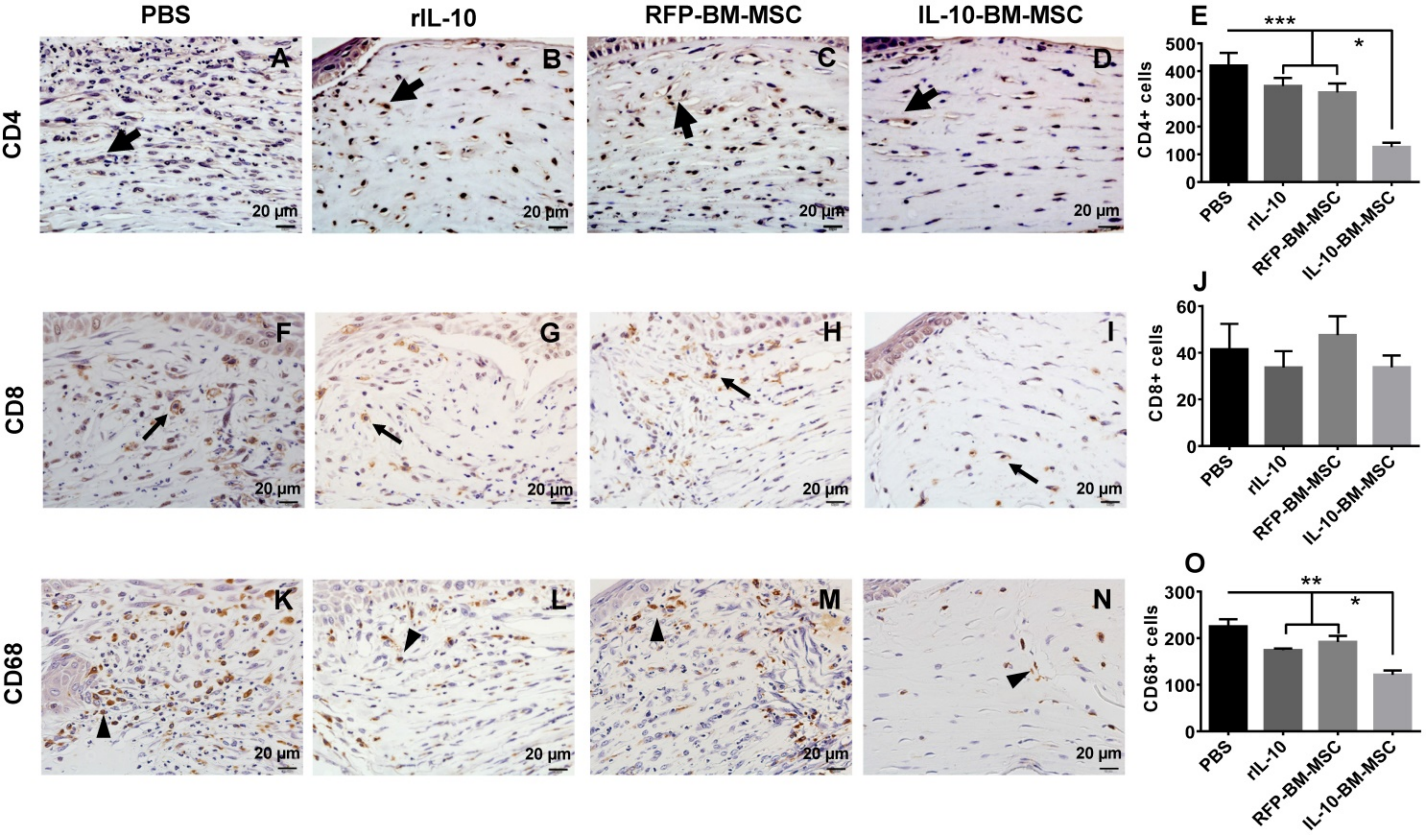
** IL-10-BM-MSCs inhibited infiltration of CD4^+^ and CD68^+^ immune cells.** Representative pictures of immunohistochemistry staining of CD4 (**A-D**), CD8 (**F-I**), and CD 68 (**K-N**) in corneal allografts were shown. Thick arrows referred to CD4^+^ cells, thin arrows CD8^+^ cells, and arrow heads CD68^+^ cells. The numbers of the cells positive for CD4 (**E**), CD8 (**J**), and CD68 (**O**) staining were quantified and compared among experimental groups (n = 6 / group). *p < 0.05, **p < 0.01, ***p < 0.001. Scale bar = 20 µm.

To investigate whether subconjunctival injections of IL-10-BM-MSCs inhibited the infiltration of T cells and macrophages, immunohistochemistry was performed to examine the infiltrating CD4^+^, CD8^+^, and CD68^+^ cells in the corneal grafts of all groups at Day 10 post transplantation. The results showed massive infiltration of CD4^+^ and CD68^+^ cells in PBS group (Figure 4A and K). The infiltration of both cell types was moderately reduced in rIL-10 and RFP-BM-MSC groups (Figure 4B, C, E, L, M, and O; for CD4, both p < 0.001, PBS vs RFP-BM-MSC, PBS vs rIL-10; CD68, both p < 0.01, PBS vs RFP-BM-MSC, PBS vs rIL-10), which was further dramatically decreased in IL-10-BM-MSC-treated group. (Figure 4D, E, N, and O; CD4, both p < 0.05, RFP-BM-MSC vs IL-10-BM-MSC, rIL-10 vs IL-10-BM-MSC; CD68, both p < 0.05, RFP-BM-MSC vs IL-10-BM-MSC, rIL-10 vs IL-10-BM-MSC). These results implicate that IL-10-BM-MSCs protect against corneal allograft rejection by inhibiting the infiltration of CD4^+^ T cells and CD68^+^ macrophages, which may serve as effectors and APCs, respectively, in the immune responses mediating allograft rejection. No statistical significance was found in the number of CD8^+^ cells infiltrating corneal grafts among experimental groups, although a trendy reduction was observed in the IL-10-BM-MSCs group (Figure 4F-J; p = 0.587, *F* = 0.659), indicating that CD8^+^ T cells might not be the predominant immune cell type regulated by the IL-10-BM-MSCs in our corneal allograft rejection model.

### Subconjunctival injections of IL-10-BM-MSCs affected the immune cell proportions in the draining lymph nodes

**Figure 5:**
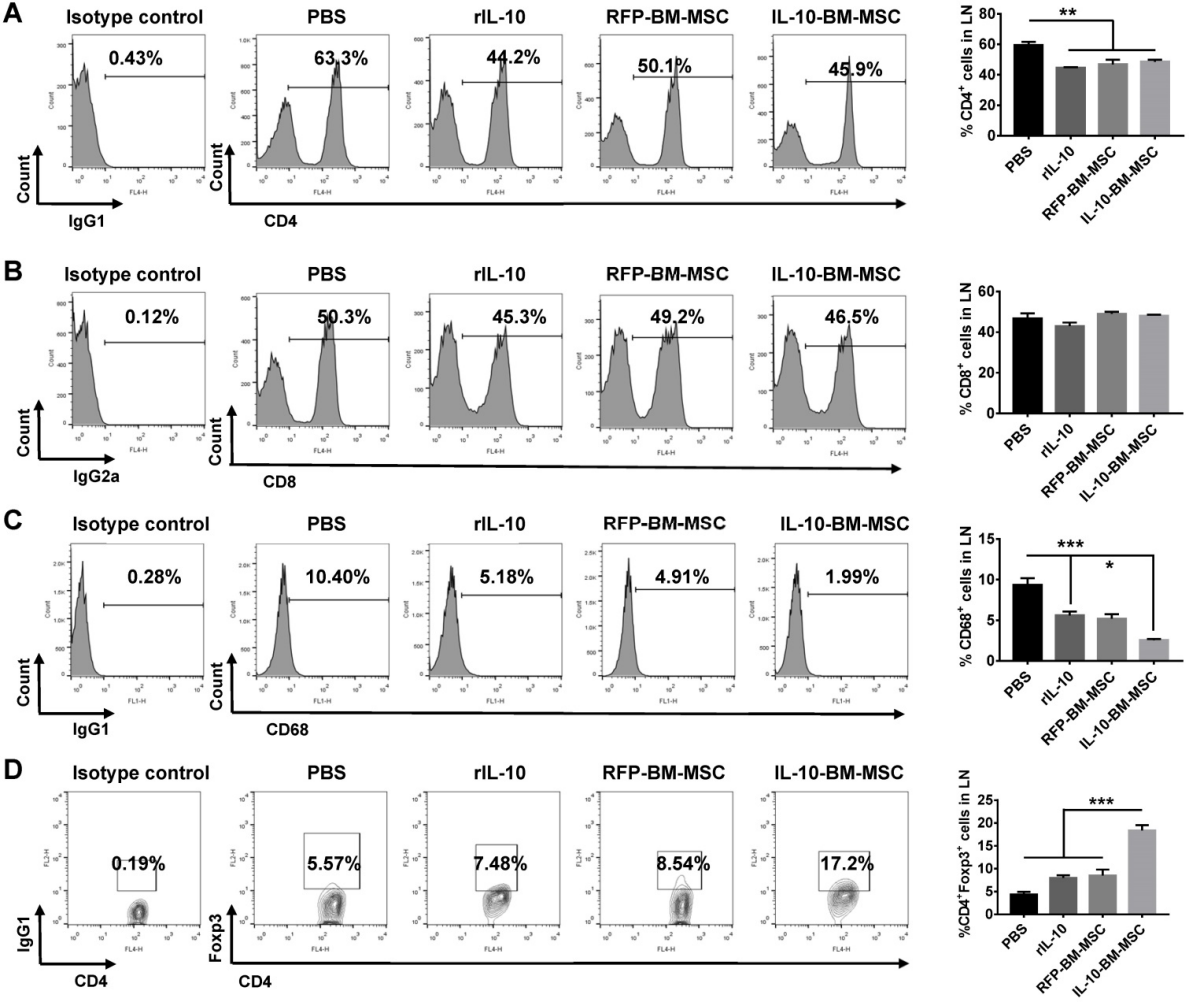
** IL-10-BM-MSC administration reduced CD4^+^ and CD68^+^ cell proportions and augmented Treg frequency in draining lymph nodes.** Representative flow cytometric histograms and quantification of CD4^+^ (**A**), CD8^+^ (**B**) and CD68^+^ (**C**) immune cells in the ocular surface draining lymph nodes at day 10 post transplantation. (**D**) Representative flow cytometric contour plots and quantified bar graph of CD4^+^Foxp3^+^ Tregs in the ocular surface draining lymph nodes at day 10 post transplantation. n=5 / group, *p < 0.05, ***p < 0.001.

To explore whether local administration of IL-10-BM-MSCs affects the immune cell proportions in the draining lymph nodes, flow cytometry was employed. The percentage of CD4^+^ T cells in PBS group was 59.46 ± 4.68%, which was significantly reduced by the subconjunctival injections of rIL-10, RFP-BM-MSCs, and IL-10-BM-MSCs (Figure 5A, all p < 0.01, PBS vs rIL-10, PBS vs RFP-BM-MSC, and PBS vs IL-10-BM-MSC), but the percentages of CD4^+^ T cells in the draining lymph nodes between the three treatment groups were not significantly different (Figure 5A, all p > 0.05, rIL-10 vs RFP-BM-MSC, RFP-BM-MSC vs IL-10-BM-MSC, rIL-10 vs IL-10-BM-MSC). Furthermore, the frequency of CD68^+^ cells, indicative of antigen-presenting macrophages, was significantly reduced, as compared to that in PBS group, in rIL-10 and RFP-BM-MSC groups (Figure 5C, both p < 0.001, PBS vs rIL-10, PBS vs RFP-BM-MSC), which was further decreased in IL-10-BM-MSC-treated group (Figure 5C, p < 0.05, rIL-10 vs IL-10-BM-MSC). By contrast, the percentages of CD8^+^ immune cells in these experimental groups did not exhibit any significant differences (Figure 5B, F=2.781, p=0.0747). Most interestingly, the CD4^+^Foxp3^+^ Tregs in the draining lymph nodes of ocular surface were enriched 3 fold following the IL-10-BM-MSC treatment (p <0.001, PBS vs IL-10-BM-MSC). These results suggested that subconjunctival injections of IL-10-BM-MSCs post corneal transplantation may reduce CD4^+^ and CD68^+^ immune cells as well as boost Treg population, whereby indicates the cellular mechanism underlying the protective effects of the locally-administered IL-10-BM-MSCs on corneal allografts.

### Tracking of the subconjunctivally-injected IL-10-BM-MSCs

To understand the homing of IL-10-BM-MSCs after subconjunctival injections, immunofluorescence staining showed that the RFP expressed by the IL-10-BM-MSCs were detectable in the conjunctival sac of the injected eye at day 10 post transplantation (Supplementary Figure 2A, C), whereas the staining using the isotype control primary antibody did not generate any positive signal (Supplementary Figure 2D, F). The subconjunctivally injected BM-MSCs were shown to reside in the conjunctival sac of the ipsilateral eye at 3 and 7 d post corneal transplantation in our previous study 16. Collectively, these results indicate that the IL-10-BM-MSCs subconjunctivally injected after keratoplasty might stay and function in the local environment of ocular surface for at least 10 d.

### IL-10-BM-MSC treatments upregulated expression of lncRNA 003946 in cornea

**Figure 6:**
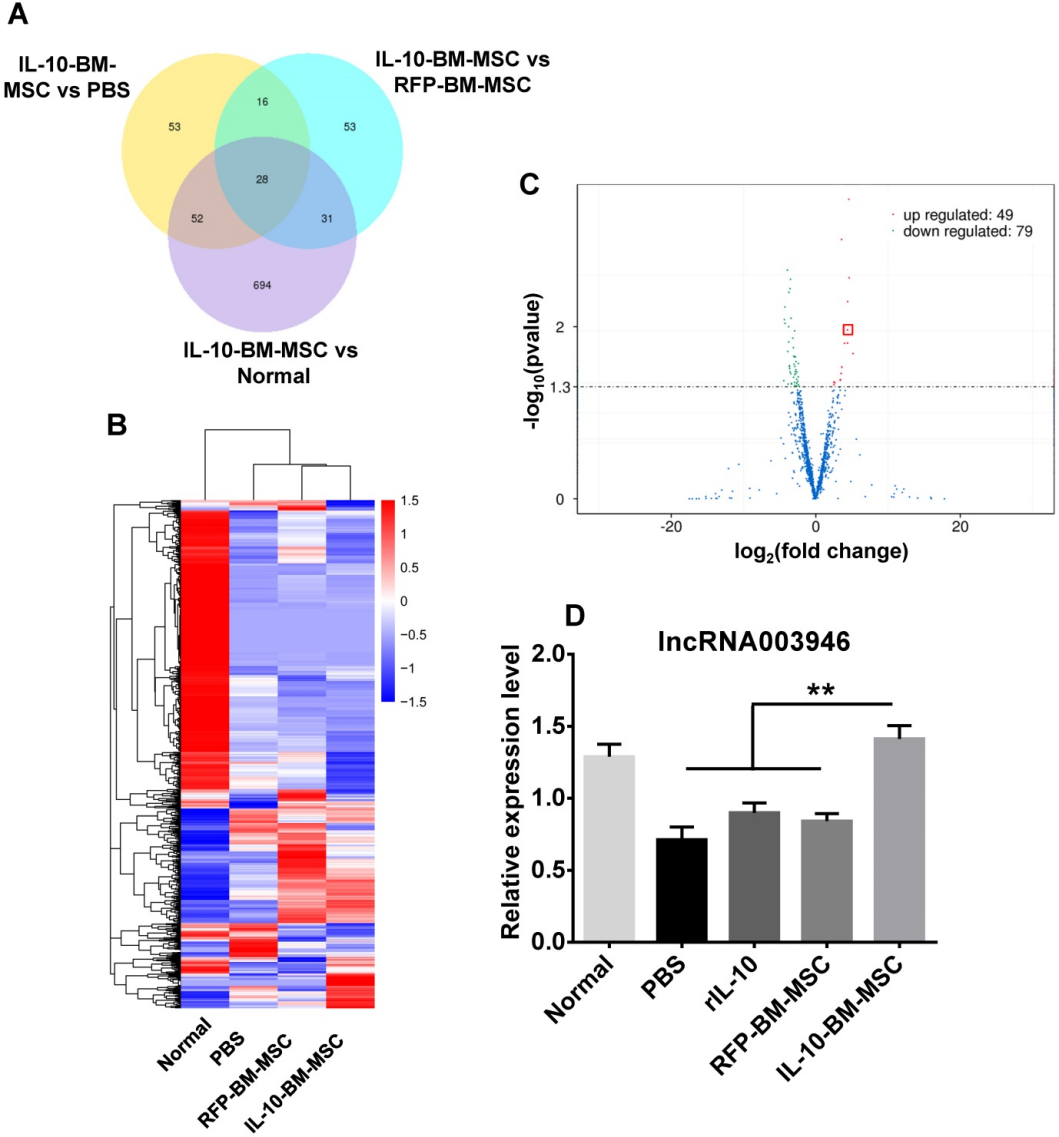
** High-throughput RNA sequencing revealed the differential lncRNA expression in the corneal allografts treated with IL-10-BM-MSCs.** (**A**) Venn diagram indicated the number of differentially expressed lncRNAs in IL-10-BM-MSC group compared with RFP-BM-MSC, PBS, and normal groups (n = 3 / group). (**B**) Hierarchical clustering of lncRNAs in the four experimental groups (n = 3 / group). (**C**) Volcanic distribution of lncRNAs. The green and red dots indicate significantly downregulated and upregulated lncRNAs, respectively (p < 0.05 with more than 2 fold changes). The red rectangle indicates the position of lncRNA 003946. (**D**) The relative expression levels of lncRNA 003946 in the cornea or corneal allografts were validated by qPCR (n = 9 / group). **p* <* 0.01.

To determine the molecular mechanism by which IL-10-BM-MSC administration suppressed allograft rejection, the corneas or corneal grafts and corresponding beds from normal (without corneal transplantation and MSC treatments), PBS, RFP-BM-MSC, and IL-10-BM-MSC groups were harvested and subjected to high-throughput RNA sequencing. The data of RNA sequencing were of high quality and reliable (Table 1). The number of differentially expressed lncRNAs was shown in Figure 6A when the transcriptome of IL-10-BM-MSC group was compared with those of normal control, PBS, and RFP-BM-MSC groups. Hierarchical clustering analysis distinguished the lncRNA expression profiling of IL-10-BM-MSC-treated group from those of normal control, PBS, and RFP-BM-MSC groups (Figure 6B). Indeed, a volcano plot showed that as compared to the RFP-BM-MSC group, the expression of 49 lncRNAs in the IL-10-BM-MSC group was upregulated, 79 downregulated (Figure 6C). Among these lncRNAs, the abundance of lncRNA 003946 was boosted 20.80 fold. This lncRNA was one of the top upregulated lncRNAs, ranking the 4^th^ according to fold changes and the 5^th^ according to the p value (Table S1). Although it was not the most upregulated one, its predicted mRNA targets were closely related to immune regulation and T cell activity (Table S1). Therefore, lncRNA 003946 was selected as the molecular target on which IL-10-BM-MSCs may act to inhibit corneal allograft rejection.

The expression profiling of lncRNA 003946 among experimental groups was verified by quantitative real-time PCR (qPCR). The expression levels of lncRNA 003946 in the corneal allografts in PBS group were reduced to 55.29% of the normal levels (Figure 6D, p < 0.01, normal vs PBS). Treatments with rIL-10 and RFP-BM-MSCs mildly upregulated lncRNA 003946 expression as compared to the PBS group (Figure 6D, p = 0.224, rIL-10 vs PBS; p = 0.193, RFP-BM-MSCs vs PBS). By contrast, IL-10-BM-MSC local administration significantly normalized the level of lncRNA 003946 (Figure 6D, both p < 0.01, rIL-10 vs IL-10-BM-MSC, RFP-BM-MSC vs IL-10-BM-MSC; p = 0.338, normal vs IL-10-BM-MSC). These results suggest that rIL-10 and BM-MSCs may act coordinately to upregulate lncRNA 003946 expression, and also implicate that IL-10-BM-MSCs may substantially promote corneal allograft survival through normalizing the expression level of lncRNA 003946.

### Screening the most efficient shRNA against lncRNA 003946 *in vivo*

Three shRNAs against lncRNA 003946 were designed and cloned into lentiviral vectors. The concentrated viruses were prepared. To screen the most effective shRNA to knockdown lncRNA 003946, lentiviral particles carrying the three shRNAs were subconjunctivally injected into the normal recipient rats. At 5 d after injection, immunofluorescence showed that *EGFP* reporter gene on the viral vector was expressed across the cornea, particularly in the epithelia and endothelia (Supplementary Figure 3A), indicating an efficient viral transduction and transgene expression. Moreover, the intact corneal layers revealed by DAPI staining suggested the structural integrity following viral transduction. The lncRNA 003946 expression levels were analyzed by qPCR at 15 d after injection. The scramble control did not alter the levels of lncRNA 003946 (Supplementary Figure 3B, p = 0.839, normal vs scramble). The corneas treated with the viral particles containing shRNA1 and 2 only demonstrated trendy reductions in the lncRNA 003946 levels (Supplementary Figure 3B, p = 0.420, normal vs shRNA1; p = 0.054, normal vs shRNA2); whereas the virus carrying shRNA3 significantly reduced the transcript level of lncRNA 003946 to 48.84% of the normal level (Supplementary Figure 3B, p < 0.05, normal vs shRNA3). Therefore, shRNA3 was selected to silence lncRNA 003946 in the next experiment.

### Knocking down lncRNA 003946 abrogated the protective effects of IL-10-BM-MSCs on corneal allografts

**Figure 7:**
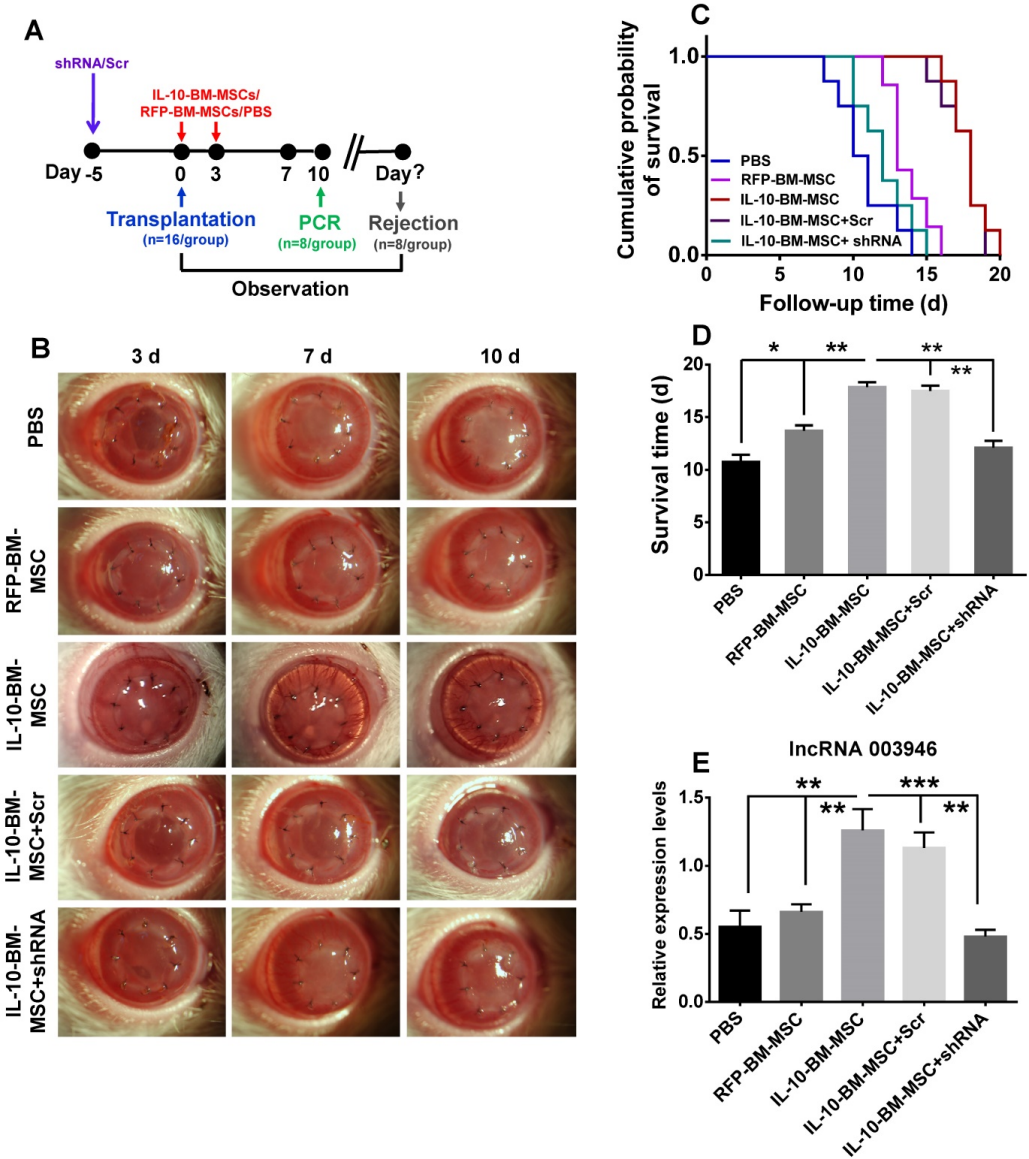
** LncRNA 003946 knockdown abrogated the protective effects of IL-10-BM-MSCs on corneal allografts.** (**A**) Schematic illustration of IL-10-BM-MSC injections in the corneal allograft rejection rats pre-treated with the selected shRNA against lncRNA 003946. (**B**) Representative pictures of corneal allografts at 3, 7, and 10 d following transplantation. (**C**) Kaplan-Meier survival curves depicted the trends of allograft survival in experimental groups (n = 8 / group). (**D**) The mean survival time of the corneal allografts was compared among the experimental groups (n = 8 /group). (**E**) The relative expression levels of lncRNA 003946 in corneal allografts and recipient beds were examined by qPCR (n= 8 / group). *p < 0.05, **p < 0.01, ***p < 0.001.

To investigate whether IL-10-BM-MSCs promoted allograft survival *via* upregulating lncRNA 003946, the lentiviral particles carrying shRNA3 or scramble were subconjunctivally injected into the recipient rats at 5 d prior to transplantation; then the model of corneal transplantation was established and RFP-BM-MSCs and IL-10-BM-MSCs were injected (Figure 7A). The corneal allografts were closely monitored (Figure 7B). The IL-10-BM-MSCs boosted the corneal allograft survival time to 17.88 ± 1.25 d as expected (Figure 7C and D, p < 0.001, PBS vs IL-10-BM-MSC). Importantly, pre-delivery of shRNA3, but not the scramble, abolished the survival-promoting effects of IL-10-BM-MSCs (Figure 7C and D, p < 0.01, IL-10-BM-MSC vs shRNA+IL-10-BM-MSC; p < 0.01, Scr+IL-10-BM-MSC vs shRNA+IL-10-BM-MSC), rendering the allograft survival time similar to that in PBS group (Figure 7C and D, p = 0.169, PBS vs shRNA+IL-10-BM-MSC). In the meanwhile, the expression pattern of lncRNA 003946 paralleled the trend of allograft survival time (Figure 7E). Specifically, the transcript levels of the lncRNA were slightly increased in the RFP-BM-MSC-treated corneas (p = 0.395, PBS vs RFP-BM-MSC). IL-10-BM-MSC treatments dramatically upregulated lncRNA 003946 expression (both p < 0.01, PBS vs IL10-BM-MSC; RFP-BM-MSC vs IL10-BM-MSC), yet this upregulation could be prevented by the shRNA against the lncRNA (p < 0.001, IL10-BM-MSC vs shRNA+IL10-BM-MSC). These results suggested that the upregulation of lncRNA 003946 is necessary for IL-10-BM-MSCs to fulfill the survival-promoting function in corneal allografts.

### Overexpression of lncRNA 003946 in cornea suppressed allograft rejection

**Figure 8:**
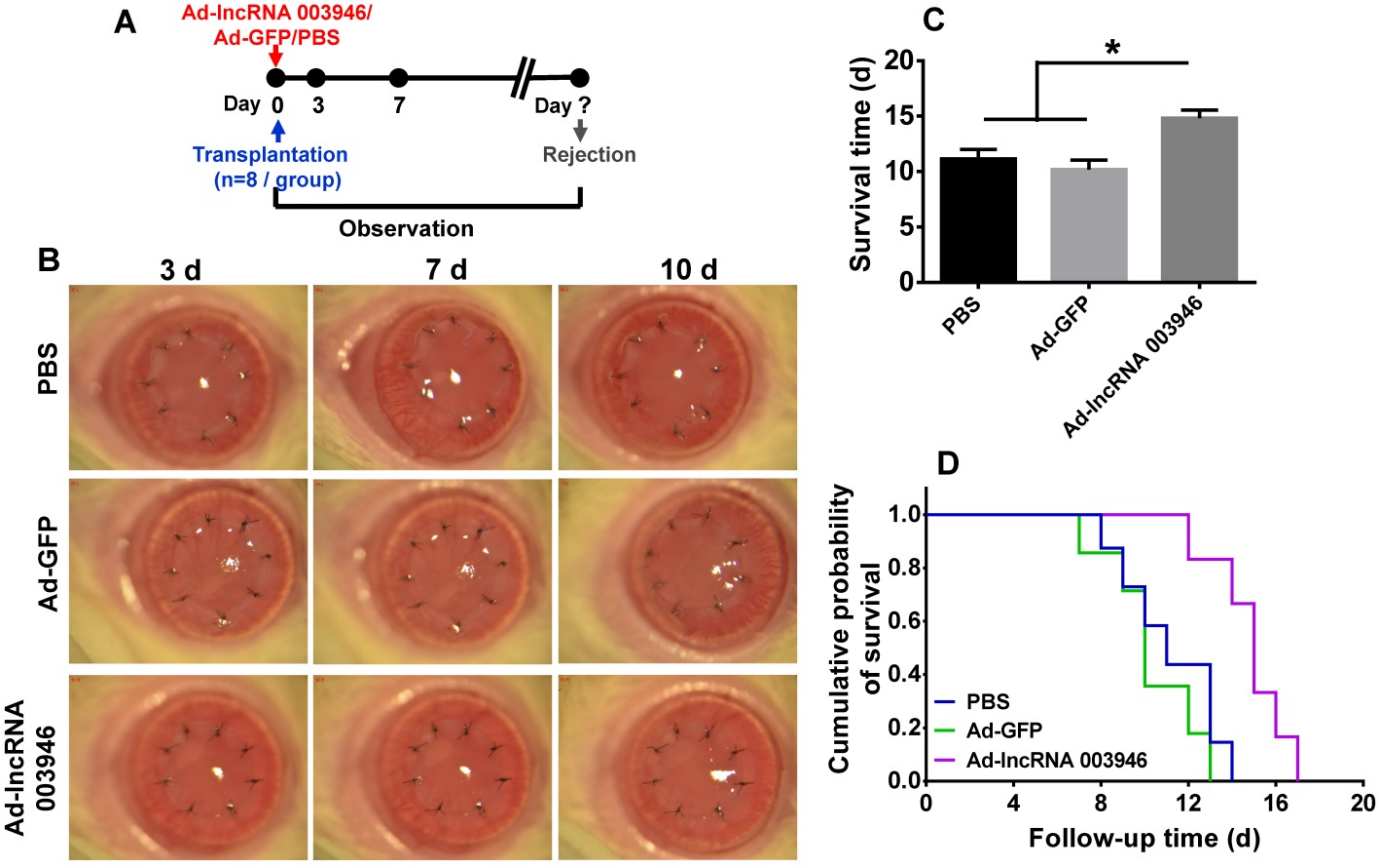
** Overexpression of lncRNA 003946 suppressed allograft rejection.** (**A**) Schematic illustration of an adenovirus injection in the cornel allograft rejection model. (**B**) Representative pictures of corneal allografts at 3, 7, and 10 d following penetrating keratoplasty. (**C**) Comparison of the mean survival time of corneal allografts among experimental groups (n = 8 / group). (**D**) Kaplan-Meier survival curves of corneal allografts in experimental groups (n = 8 / group). * p < 0.05.

To further verify the effect of lncRNA 003946 on corneal allograft survival, the corneal allograft transplantation model was established, and the adenovirus carrying lncRNA 003946 (Ad-lncRNA 003946) or the adenovirus carrying GFP (Ad-GFP) was subconjunctivally injected immediately after transplantation (Figure 8A). The corneal allografts in PBS group and Ad-GFP group were similar, being edematous and opaque on day 7 and 10 post transplantation. By contrast, the allografts in Ad-lncRNA 003946 group were significantly alleviated in edema and opacity at the both time points (Figure 8B). The survival time of the allografts in Ad-lncRNA 003946 group was significantly longer than PBS and Ad-GFP groups (Figure 8C and D, both p < 0.05, for PBS vs Ad-lncRNA 003946, Ad-GFP vs Ad-lncRNA 003946; p > 0.05 for PBS vs Ad-GFP).

### IL-10-BM-MSC treatments upregulated lncRNA 003946 expression in CD68^+^ infiltrating cells

**Figure 9:**
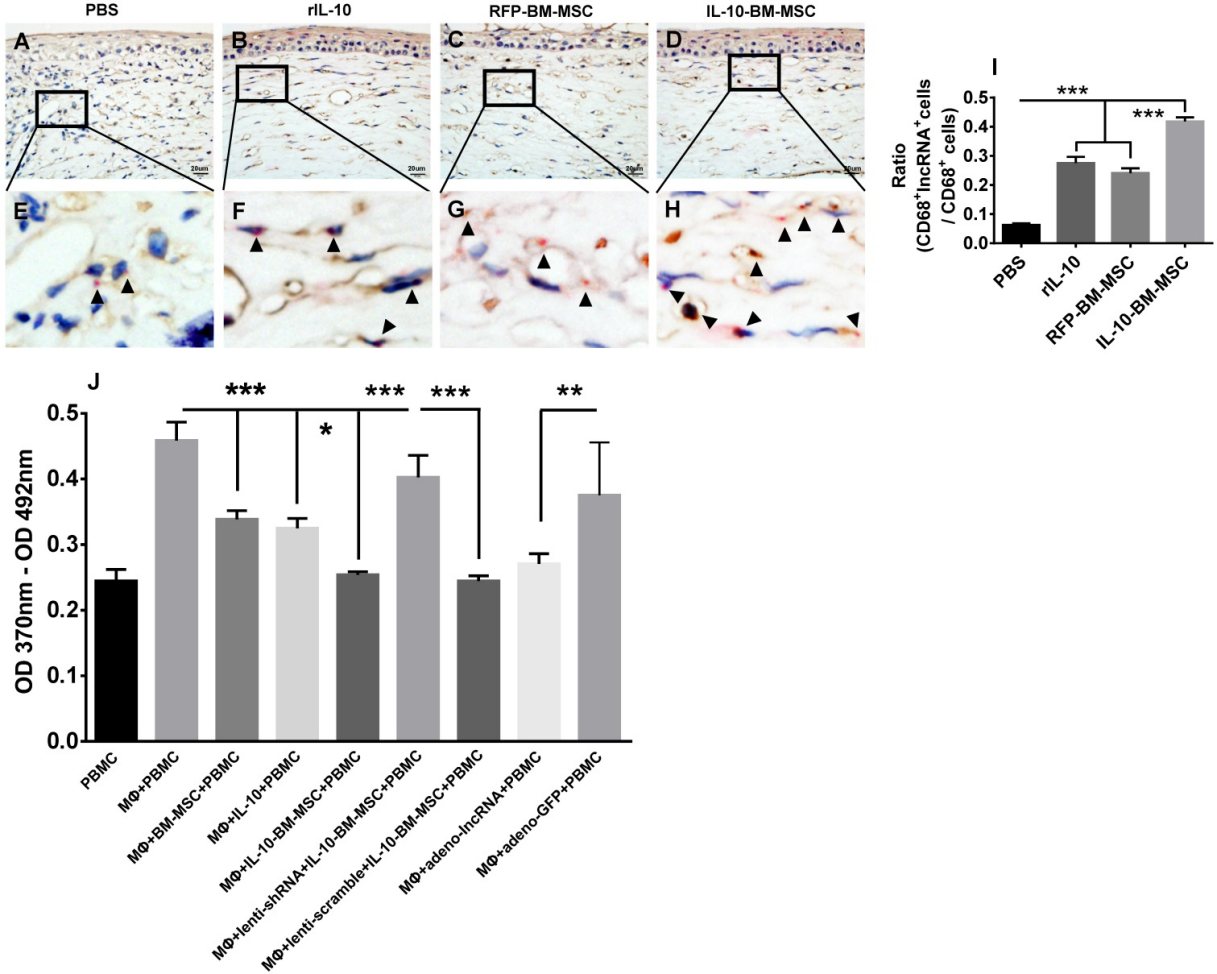
** Upregulation of lncRNA 003946 expression in CD68^+^ macrophages and its inhibition on the antigen-presenting capability of macrophages.** The representative pictures of CD68 (brown) and lncRNA 003946 (red) staining in corneal allografts of PBS (**A**), rIL-10 (**B**), RFP-BM-MSC (**C**), and IL-10-BM-MSC (**D**) groups were shown. The magnified portions of these pictures were also shown in (**E-H**). The arrow heads indicate positive signals for lncRNA 0003946. The ratios of CD68^+^lncRNA 003946^+^cells over CD68^+^ cells were quantified and compared among groups (**I**). The proliferation of PBMCs in presence of macrophages under various conditions was examined by a BrdU assay and shown in (**J**). n=6 / group, *p < 0.05, **p < 0.01, ***p < 0.001. Scale bar = 20 µm. PBMC: peripheral blood mononuclear cell; MΦ: macrophage

To ascertain the cell type where lncRNA 003946 is predominantly expressed and how the cell expression profiling of this lncRNA affects corneal allograft rejection, RNAscope *in situ* hybridization was conducted. The results showed that lncRNA 003946 was mainly expressed in the CD68^+^ immune cells infiltrating the corneal allografts. In PBS group, few CD68^+^ cells expressed lncRNA 003946 (Figure 9A, E, and I); whereas in rIL-10 and RFP-BM-MSC groups, the percentages of CD68^+^ cells that co-express lncRNA 003946 were significantly increased as compared to PBS group (Figure 9B, C, F, G, and I, both p < 0.001, PBS vs RFP-BM-MSC, PBS vs rIL-10). More importantly, the percentages of CD68^+^lncRNA 003946^+^ cells were significantly enhanced in IL-10-BM-MSC-treated group (Figure 9D, H, and I, both p < 0.001, RFP-BM-MSC vs IL-10-BM-MSC, rIL-10 vs IL-10-BM-MSC). These results demonstrated that IL-10-BM-MSCs elevated the positivity of lncRNA 003946 expression in CD68^+^ immune cells, implying that the IL-10-BM-MSC's protection on corneal allograft survival might be associated with the lncRNA 003946 upregulation in the infiltrating CD68^+^ immune cells.

### LncRNA 003946 inhibited antigen presentation of CD68^+^ macrophages

The infiltrating CD68^+^ immune cells are the antigen-presenting macrophages. Therefore, the macrophages were isolated from bone marrow and their capabilities to promote T lymphocyte proliferation under various conditions were examined by a BrdU assay. The results demonstrated that BM-MSC, rIL-10, and IL-10-BM-MSC all significantly inhibited the proliferation of the T cells isolated from the corneal allograft rejection model and cultured with bone marrow-derived macrophages, with IL-10-BM-MSC exhibiting the strongest inhibition among the three groups (Figure 9J, all p < 0.001, for MΦ + BM-MSC + PBMC vs MΦ + PBMC, MΦ + IL-10 + PBMC vs MΦ + PBMC, MΦ + IL-10-BM-MSC + PBMC vs MΦ + PBMC; both p < 0.05 for MΦ + BM-MSC + PBMC vs MΦ + IL-10-BM-MSC + PBMC, MΦ + IL-10 + PBMC vs MΦ + IL-10-BM-MSC + PBMC). Moreover, the inhibitory effect of IL-10-BM-MSC on T cell proliferation was abrogated by infecting the CD68^+^ macrophages with the lentivirus carrying shRNA3 against lncRNA 003946, but not by the lentivirus carrying scramble sequence (Figure 9J, p < 0.001, MΦ + IL-10-BM-MSC + PBMC vs MΦ + lenti-shRNA + IL-10-BM-MSC + PBMC, MΦ + lenti-scramble + IL-10-BM-MSC + PBMC vs MΦ + lenti-shRNA + IL-10-BM-MSC + PBMC). Conversely, infecting the macrophages with the adenovirus overexpressing lncRNA 003946, but not the adenovirus only expressing GFP, mimicked the suppressing effect of IL-10-BM0MSCs on T cell proliferation (Figure 9J, p < 0.01, MΦ + PBMC vs MΦ + ad-lncRNA + PBMC, MΦ + ad-lncRNA + PBMC vs MΦ + ad-GFP + PBMC). These results suggested that IL-10-BM-MSCs may inhibit T cell proliferation through upregulating lncRNA 003946 expression in the CD68^+^ antigen-presenting macrophages.

## Discussion

In the present study, we investigated the protective effects of *IL-10* gene-modified BM-MSCs on allograft survival and explored the underlying mechanisms in a well-established rat model of corneal allograft rejection. Our data showed that the genetically-engineered BM-MSCs could constitutively secret high-level IL-10, and the lentivirus-mediated genetic manipulation did not alter their self-renewal and multi-differentiation potentials, on top of which their immunosuppressive function was profoundly enhanced. Furthermore, subconjunctival administration of IL-10-BM-MSCs substantially ameliorated the opacity and edema, doubled the survival time of corneal allografts, and dramatically suppressed the infiltration of CD4^+^ T cells and CD68^+^ macrophages. These previously undescribed protective effects strongly support the possibility of developing the genetically-engineered IL-10-secrecting BM-MSCs into an effective modality counteracting corneal allograft rejection. As for the mechanism, we subjected the IL-10-BM-MSC-treated corneas and recipient beds to high-throughput RNA sequencing, and for the first time, linked the mechanism of action of the genetically-engineered BM-MSCs to lncRNA. Specifically, lncRNA 003946 expression was dramatically upregulated after treatment with IL-10-BM-MSCs in comparison to that following treatment of rIL-10 or empty vector-transduced BM-MSCs. Moreover, silencing lncRNA 003946 by shRNA completely abolished, whereas overexpressing lncRNA 003946 mimicked the protection of corneal allografts endowed by IL-10-BM-MSCs. Subsequently, we ascertained that the IL-10-BM-MSCs upregulated the expression of lncRNA 003946 predominantly, if not completely, in the infiltrating CD68^+^ macrophages. Furthermore, the results of an *in vitro* assay suggested that IL-10-BM-MSCs exerted the greatest suppressing effects on the antigen-presenting capability of macrophages; knocking down or overexpressing lncRNA 003946 in the macrophages abrogated or mimicked the effects of IL-10-BM-MSCs on antigen presentation. Taken together, these results suggest that the genetically-engineered IL-10-expressing BM-MSCs protect against corneal allograft rejection *via* upregulating lncRNA 003946 in CD68^+^ antigen-presenting macrophages; lncRNA 003946 and the infiltrating antigen-presenting macrophages are the molecular and cellular targets upon which IL-10-BM-MSCs may act.

Recent studies demonstrate that systemic administration of MSCs promotes allograft survival in corneal allograft rejection models. For instance, Oh et al 33 have shown that intravenous administration of MSCs prolongs survival time of corneal allografts. Omoto and colleagues 17 demonstrated that systemically-delivered MSCs improved survival of corneal transplants by inhibiting APC maturation and T cell induction, which is consistent with our previous study 15. On the other hand, local delivery of MSCs has advantages, including reduced cell volume, increased concentration of MSCs at target tissue, and diminished tumorigenicity 16. Indeed, our data from the current study showed prolonged allograft survival and alleviated corneal opacity and edema after subconjunctival injections of BM-MSCs. Further study indicated that the BM-MSCs exert the protective effects, at least partially, by generating the anti-inflammatory factor, IL-10. However, local administration of plain BM-MSCs offered limited extension to the survival time of corneal allograft (only 2 d). New approach to improve efficacy of plain BM-MSCs, such as genetic modification, is thus considered.

IL-10, as a potent suppressor of inflammation, facilitates immune tolerance after organ transplantation 34, 35. It inhibits maturation of APCs and activation of monocyte-/macrophage-dependent T cells. Therefore, the anti-inflammatory and immunomodulatory properties of IL-10 may be combined with the advantages of local BM-MSC administration to augment the MSC's protective function on corneal allograft survival. Moreover, BM-MSCs may serve as an excellent delivery vehicle due to its low immunogenicity and homing capability towards injury or inflammation site 36, 37. As a matter of fact, in this study, subconjunctival administration of the genetically-modified IL-10-overexpressing BM-MSCs after corneal transplantation alleviated the allograft edema and opacity and prolonged the survival time more effectively than plain BM-MSCs. This is a proof-of-principle study that supports to further develop IL-10-BM-MSCs into an efficacious interventional modality to corneal transplantation rejection. In addition, the synergistic or additive interaction between IL-10 and MSCs has been explored in lung allotransplantation, where IL-10-expressing MSCs were administered prior to transplantation 19. In the current study, we did observe a positive interaction between IL-10 and BM-MSCs in promoting corneal allograft survival and inhibiting immune cell infiltration. However, we did not test the effects of IL-10-BM-MSC pre-treatment, since the results of our previous study suggested that prophylactic administration of BM-MSCs may cause accelerated immune rejection 16.

We next sought to probe the molecular mechanism underlying IL-10-BM-MSCs' protective effects on allograft survival. Some lncRNAs, such as lincRNA-Cox2 and Lethe, are crucial inflammatory mediators and can be induced by anti-inflammatory factors 30, 38. Moreover, Geng et al 39 reported that IL-22, a member of IL-10 family, induced the expression of lncRNA H19 to promote mucosal regeneration, suggesting that a lncRNA might serve as a downstream mediator of a Th2 cytokine. Indeed, in this study, high-throughput RNA sequencing analysis demonstrated dramatic upregulation of lncRNA 003946 expression after IL-10-BM-MSC treatments as compared to plain BM-MSC or rIL-10 treatments; whereas silencing and ovexpressing this lncRNA abolished and simulated the protective effects of IL-10-BM-MSCs on the corneal allografts. These results serve as the first line of evidence suggesting a novel lncRNA as a downstream molecular target of IL-10-BM-MSCs.

The next question would be which cell type harboring the increased abundance of lncRNA 003946 mediates the suppressing effects of IL-10-BM-MSCs on corneal allograft rejection. In view of the causal role of APCs in corneal allograft rejection 40, 41, the graft-infiltrating and draining-lymph-node-residing CD68^+^ macrophages, whose number has been synergistically or additively suppressed by IL-10-BM-MSCs in comparison to rIL-10 or plain BM-MSCs, would be the best candidate. Indeed, the RNAscope *in situ* hybridization revealed that the expression of lncRNA 003946 was significantly upregulated in the CD68^+^ APCs infiltrating in the corneal allografts of the rIL-10 and RFP-BM-MSC groups, which was even enhanced in the IL-10-BM-BMC-treated corneal allografts. Further, an *in vitro* assay using the co-cultures of isolated peripheral blood mononuclear cells (PBMCs) and macrophages proved that knocking down lncRNA 003946 in the macrophages negated the suppressing effects of IL-10-BM-MSCs on the macrophages' antigen-presenting capability, thereby promoting the T lymphocyte proliferation. On the other hand, overexpressing the lncRNA in the macrophages mimicked the IL-10-BM-MSCs' suppressing effects, inhibiting the macrophages' antigen presentation and T cell proliferation. These results indicate that at least the graft-infiltrating, CD68^+^ antigen-presenting macrophages upregulated lncRNA 003946 expression in response to IL-10-BM-MSC local administration, and that the upregulated lncRNA then suppressed antigen presentation and effector T cell proliferation.

Moreover, Gene Ontology (GO) and Kyoto Encyclopedia of Genes and Genomes (KEGG) pathway enrichment analyses predicted 13 gene transcripts as the potential downstream targets of lncRNA 003946 (Table S1). It would be interesting in the future experiments to ascertain the molecular pathway through which the upregulated lncRNA 003946 in macrophages suppresses antigen presentation, which, in turn, subdues immune rejection and sustains allograft survival following corneal transplantation.

In conclusion, lentivirus-mediated IL-10 overexpression in BM-MSCs enhanced the MSCs' immune inhibitory capability while maintaining self-renewal and multi-differentiation potentials. Subconjunctival injections of IL-10-BM-MSCs remarkably protected corneal allografts from immune rejection and prolonged allograft survival in a rat model of corneal allograft rejection. The mechanism underlying such protection involves upregulation of a novel lncRNA in CD68^+^ APCs.

## Materials and Methods

### Animals

Three hundred and ten Lewis female rats (8 weeks of age, body weight 180 - 200 g) and 130 Wistar female rats (8 weeks of age, body weight 180 - 200 g) were purchased from Beijing Vital River Laboratory Animal Technology Co., Ltd. (Beijing, China). The rats were housed at a temperature- and humidity-conditioned room (25 ± 1 ºC with the relative humidity of 40 - 70%) and fed *ad lib* with food and water. The illumination was under 12 h light-dark cycles. All experimental procedures were approved by the Institutional Animal Care and Use Committee of Tianjin Medical University (permission number: SYXK2009-0001) and in accordance with the Guide for the Care and Use of Laboratory Animals published by the U.S. National Institutes of Health (NIH; Publication No. 85-23, revised 1996).

### BM-MSC cultures

OriCell Wistar rat MSCs (Cat.^#^ RAWMX-01001, Cyagen, Santa Clara, CA, USA), derived from bone marrow of Wistar rats, were cultured in 25 cm^2^ tissue culture flasks (Corning, Corning, NY, USA) under 37 ºC in a 5% CO_2_ humidified incubator. The BM-MSCs were maintained in the OriCell MSC growth media (Cat.^#^ GUXMX-90011, Cyagen, Santa Clara, CA, USA), consisting 88% OriCell MSC basal media, 10% MSC-qualified fetal bovine serum (Cyagen, Santa Clara, CA, USA), 1% penicillin-streptomycin (Cyagen, Santa Clara, CA, USA), and 1% Glutamine (Cyagen, Santa Clara, CA, USA). The cells were passaged at 1:3 upon 90% confluency. The BM-MSCs at passage 3 with optimal stability and multi-differentiation capability were used for lentivirus transduction.

### Penetrating keratoplasty

Orthotopic penetrating keratoplasty was performed on the right eyes of recipient rats by an experienced ophthalmologist as described previously 16. In brief, both host Lewis rats and donor Wistar rats were anesthetized by an intraperitoneal injection of 1% pentobarbital sodium (0.6 ml / 100 g). The recipient's pupils were dilated by 0.5% tropicamide. The graft was obtained from the central cornea of the donor using a 3.5-mm trephine, and secured on the recipient bed (3 mm in diameter) with 8 interrupted 10-0 nylon sutures. The endothelia were protected with care during operation.

### BM-MSC administration

To investigate the effects of BM-MSCs on allograft survival, the Lewis rats were randomly divided into PBS group (n = 20) and BM-MSC group (n = 20) after corneal transplantation. The BM-MSC group was subconjunctivally injected with 2 × 10^6^ BM-MSCs suspended in 100 μl PBS immediately (day 0) and at 3 d (day 3) after transplantation. The PBS group was injected with the same volume of PBS at both time points. The amount of cells injected and injection frequency were determined based on our previous work 16.

### Assessment of corneal allografts

All grafts were closely monitored under a slit-lamp biomicroscope on a daily basis following corneal transplantation and the pictures were taken using a Nikon D90 camera (Nikon Corporation, Tokyo, Japan) attached to the slit-lamp biomicroscope. The rejection index (RI) was calculated according to opacity (0-4), edema (0-2), and vascularization (0-4) of the grafts, as described by Larkin and colleagues 42 (Table 2). The RI ≥ 5 together with the corneal opacity score ≥ 3 indicated graft rejection 42. The evaluations of all the corneal grafts were performed by two experienced ophthalmologists unaware of grouping.

### Extraction and quantification of total protein and enzyme-linked immunosorbent assay

Total protein from the grafts and their recipient beds of PBS and BM-MSC groups (n = 8 / group) at 10 d post transplantation was extracted using a Tissue Protein Extraction Kit (CWBIO, Beijing, China). The concentration of total protein was determined by a Bicinchoninic Acid Protein Assay Kit (CWBIO, Beijing, China) following the company's instructions. The concentrations of IL-10 in the total protein samples were determined using an enzyme-linked immunosorbent assay (ELISA) kit (R&D systems, Minneapolis, MN, USA) according to manufacturer's instructions. Briefly, the protein samples were added into a 96-well microplate pre-coated by a monoclonal antibody to rat IL-10. After IL-10 in the samples bound to the pre-coated antibody, an enzyme-linked polyclonal antibody was added to sandwich the immobilized IL-10. Finally, the colorimetric substrates were added for quantification. The serially-diluted recombinant rat IL-10 was used to generate a standard curve. The standard curve could also serve as the positive controls; the wells containing only diluent were included as the negative controls. The optical density (OD) was measured by an Infinite 200 PRO Multimode Microplate Reader (Tecan Group Ltd., Männedorf, Switzerland) at the wavelength of 450 nm with the correction at 540 nm. The concentrations of IL-10 (pg/ml) in samples were calculated according to the OD value and the standard curve. The concentration of IL-10 was normalized to the total protein concentration (μg/μl).

### Lentivirus packaging, tittering, and transduction of BM-MSCs

#### Cloning of rat IL-10 gene

Total RNA was isolated from liver tissue of Lewis rat using a GeneJET RNA Purification Kit (Thermo Fisher Scientific, Waltham, MA, USA) following the manufacturer's instructions. The concentration and purity of RNA were determined by a Nanodrop 2000 (Thermo Fisher Scientific, Waltham, MA, USA). One microgram of the total RNA was reverse transcribed into cDNA using a RevertAid First Strand cDNA Synthesis Kit (Thermo Fisher Scientific, Waltham, MA, USA). *IL-10* coding sequence was amplified from the cDNA template using GoTaq Green 2X Master Mix (Thermo Fisher Scientific, Waltham, MA, USA) in a GeneAmp PCR System 2400 (PerkinElmer, Waltham, MA, USA) with specific primers (Table 3). The PCR product was purified using a GeneJET PCR Purification Kit (Thermo Fisher Scientific, Waltham, MA, USA), cloned into a TOPO TA vector (Life Technologies, Grand Island, NY, USA), confirmed by sequencing, and then subcloned into a lentiviral expression vector (CD512B-1, System Biosciences, Mountain View, CA) through *BamH I* and *Not I* sites. The resulting vector was termed as Lenti-IL-10. The empty vector was named Lenti-RFP and served as a negative control.

#### Lentivirus packaging

The self-inactivated third generation lentivirus expression system (System Biosciences, Mountain View, CA, USA) was employed to package viruses. All the lentivirus-involving experiments, including packaging, concentration, tittering, and injections, were performed in a dedicated Biosafety Level 2 microbiology laboratory, and all the experimental procedures adhered to the health and safety guidelines stated by BIOSAFETY LEVEL 2 LABORATORY PRACTICES from NIH. The packaging of lentivirus was performed as described previously 43, 44. Briefly, 293T cells were seeded on the 6-well plates (Corning, Corning, NY, USA) coated by rat tail collagen (Sigma-Aldrich, St. Louis, MO, USA) at a density of 8 × 10^5^ cells / well. Twenty-four hours later, the 293T cells were transfected by 2 μg mixture of endotoxin-free plasmids including Lenti-IL-10 or Lenti-RFP, RRE, REV, and VSVG (mass ratio 4:2:1:1.2) with the aid of chloroquine (Sigma-Aldrich, St. Louis, MO, USA) and X-tremeGENE HP DNA Transfection Reagent (Roche, Branford, CT, USA). At 48 h post transfection, the culture media were collected, filtered, and stored as unconcentrated viruses at -80 °C.

#### Lentivirus tittering

To determine the titers of the unconcentrated viruses, 293T cells were seeded at 1 × 10^6^ cells / well in 6-well culture plates (Corning, Corning, NY, USA) and transduced with 1, 10, and 100 μl of the unconcentrated lentiviruses with assistance of polybrene (Sigma-Aldrich, St. Louis, MO, USA). At 64 h post transduction, the cells were digested with trypsin and analyzed by a FACSCalibur (BD Biosciences, San Jose, CA, USA). The gate was set based on forward and side scatter characteristics of plain 293T cells. The cell population above the border was deemed positive for RFP expression and viral transduction. The titer was calculated as the averaged number of cells that can be transduced by 1 ml virus. The titer of Lenti-IL-10 was 1.47 × 10^7^ transduction units (TU)/ml, and that of Lenti-RFP 1.62 × 10^7^ TU/ml.

#### BM-MSC transduction and FACS purification

Cells were transduced as previously described 43, 44. Briefly, the titers of Lenti-IL-10 and Lenti-RFP were equally adjusted. The BM-MSCs were seeded in 6-well plates (Corning, Corning, NY, USA) at a density of 5 × 10^5^ cells / ml and transduced with either virus of equal titer in presence of polybrene (Sigma-Aldrich, St. Louis, MO, USA). Sixteen hours later, the media were replaced by 2 ml fresh complete culture media, with which the cells were cultured thereafter. At 5 d post lentiviral transduction, the transduced BM-MSCs were sorted by a FACSCalibur (BD Biosciences, San Jose, CA, USA). The purified IL-10-producing BM-MSCs were designated as IL-10-BM-MSCs and maintained in culture.

In addition, the genomic DNA of IL-10-BM-MSCs was extracted using an OMEGA E.Z.N.A Tissue DNA Kit (Solarbio, Beijing, China). The PCR was performed in a GeneAmp PCR System 2400 (PerkinElmer, Waltham, MA, USA) using 10 μl GoTaq Green 2X Master Mix (Thermo Fisher Scientific, Waltham, MA, USA), 2 μl DNA template from IL-10-BM-MSCs, and specific detection primers (Table 3) for *Woodchuck Hepatitis Virus Posttranscriptional Regulatory Element (WPRE)*. The *WPRE* sequence is only present in lentiviral expression vector. The PCR reactions using DNA from plain BM-MSCs and water as templates served as negative controls. Then the PCR products were visualized on a 1% agarose gel.

### Morphology of BM-MSCs and IL-10-BM-MSCs

The unsorted and sorted BM-MSCs following Lenti-IL-10 transduction were seeded on coverslips in a 24-well plate (Corning, Corning, NY, USA) at a density of 8 × 10^4^ cells / well. The cells were cultured at 37 °C for 24 h, washed twice with pre-warmed Dulbecco's Phosphate Buffered Saline (DPBS, Gibco, Grand Island, NY, USA), and fixed with 1% paraformaldehyde (PFA, Sigma-Aldrich, St. Louis, MO, USA) for 5 min at room temperature. Afterwards, the coverslips were mounted with the ProLong Gold Antifade with DAPI reagent (Life Technologies, Grand Island, NY, USA). The cells were observed under a fluorescence microscope (BX51, Olympus Optical Co. Ltd., Tokyo, Japan) and the representative pictures were taken by the cellSens Standard electronic system (Olympus Optical Co. Ltd., Tokyo, Japan).

### *In vitro* differentiation of BM-MSCs and IL-10-BM-MSCs

Adipogenic and osteogenic differentiation potentials of plain BM-MSCs and IL-10-BM-MSCs at 3^rd^ passage were evaluated *in vitro* following the manufacturer's instruction. All the reagents used in the differentiation assays were purchased from Cyagen US Inc. (Santa Clara, CA, USA).

#### Adipogenic differentiation

BM-MSCs and IL-10-BM-MSCs at the 3^rd^ passage were seeded in 6-well plates (Corning, Corning, NY, USA) at a density of 2 × 10^5^ cells / well. When the cells were 100% confluent, they were incubated with 2 ml adipogenic differentiation complete media A (induction media), composed of basal media A, 10% FBS, 1% penicillin-streptomycin, 1% glutamine, 2‰ insulin, 1‰ IBMX, 1‰ rosiglitazone, and 1‰ dexamethasone (Cyagen US Inc., Santa Clara, CA, USA). Three days later, the media were replaced with adipogenic differentiation media B (maintenance media) for 24 h, and then changed back to the media A. After 3 - 5 cycles of medium change, the adipogenic differentiation potential was assessed via 0.5% oil red O staining for detection of lipid droplets within the cells.

#### Osteogenic differentiation

The cells were seeded at a density of 3 × 10^5^ cells/well in 6-well tissue culture plates (Corning, Corning, NY, USA) pre-coated with 0.1% gelatin solution. The culture media were changed to osteogenic differentiation media when cells reached 60 - 70% confluence. The media were replaced with fresh osteogenic differentiation media every 2 d. After incubating with the osteogenic differentiation media for 2 - 4 w, the cells were fixed and stained with alizarin red S. Osteogenic potential was evaluated by alizarin red staining for calcified deposits.

### Growth of IL-10-BM-MSCs

BM-MSCs and IL-10-BM-MSCs (n = 3 /group) were seeded into 24-well plates (Corning, Corning, NY, USA) at a density of 4 × 10^4^ cells / ml, and cultured at 37 °C in a 5% CO_2_ humidified incubator. The cells were counted every 24 h by two experienced technicians blinded with the experimental grouping. The experiment was repeated independently for at least 3 times.

### IL-10 secretion from IL-10-BM-MSCs

The amount of IL-10 secreted by plain BM-MSCs and IL-10-BM-MSCs at the 3^rd^ passage was determined using an ELISA kit (R&D systems, Minneapolis, MN, USA). Briefly, the cells at a density of 5 × 10^5^ cells / well were seeded in 6-well plates (Corning, Corning, NY, USA) and cultured with OriCell MSC Growth Media (Cyagen, Santa Clara, CA, USA) at 37 °C in a 5% CO_2_ humidified incubator (n = 4 / group). At 48 h after seeding, the cell culture supernatants were collected following a brief centrifugation to precipitate dead cells and cell debris. The supernatants were subjected to the IL-10 ELISA as described above.

### T-cell proliferation assay

T-cell proliferation assay was performed to detect BrdU incorporation into T cells using a cell proliferation ELISA kit (Roche DGmbH, Mannheim, Germany). Briefly, 100 μl irradiated plain BM-MSCs or IL-10-BM-MSCs (15 Gy, at densities of 1 × 10^3^, 1 × 10^4^, 2 × 10^4^, 1 × 10^5^ cells / well) were seeded in 96-well culture plates (Corning, Corning, NY, USA) that had been pre-coated with anti-rat CD3 antibody (BD, Franklin Lakes, NJ, USA) and cultured at 37 °C for 12 h (n = 6 / group). The PBMCs were isolated from the blood of Wistar rats as described previously 45. Then equal volume of PBMCs were added to the 96-well plates (1 × 10^5^ cells / well) in the presence of anti-rat CD28 (0.5 mg/ml, BD, Franklin Lakes, NJ, USA). Forty-eight hours later, the PBMCs and supernatants were transferred to a new 96-well plate (Corning, Corning, NY, USA). The BrdU incorporation was then detected by an Infinite 200 PRO Multimode Microplate Reader (Tecan Group Ltd., Männedorf, Switzerland). The wells without any cell were included as blank controls, and the wells without BrdU as background controls.

### BM-MSC treatment protocol and clinical assessments

Rats subjected to penetrating keratoplasty were randomly divided into PBS (n = 26), rIL-10 (n=26), BM-MSC (n = 20), RFP-BM-MSC (n = 26), and IL-10-BM-MSC (n = 26) groups. For each stem cell-treated group, the recipient rats were all subconjunctivally injected with 2 × 10^6^ cells suspended in 100 μl PBS immediately after and at 3 d following the transplantation. In PBS group, the recipients were injected with the same volume of PBS. The cell dosage and injection timing and frequency were chosen according to the previous studies 15, 16. In rIL-10 group, the recipients received two subconjunctival injections of 2 ng rIL-10 (dissolved in 100 µl PBS; R&D systems, Minneapolis, MN, USA) in the right eyes at the same schedule. The concentration of the injected rIL-10 solution was calculated according to the amount of IL-10 secreted by 2 × 10^6^ IL-10-BM-MSCs as determined by the ELISA (Figure 3C). All the corneal grafts were monitored under a slit-lamp biomicroscope on a daily basis and the representative pictures taken as mentioned above.

### Immunohistochemistry

At 10 d post transplantation, the comparable part of the eye balls from each group (n = 6 / group) were paraffin sectioned (5 µm / section). Eight sagittal sections from each eye ball were prepared for immunohistochemistry. Briefly, following deparaffinization, rehydration, and heat-mediated antigen retrieval, the sections were incubated with rabbit anti-rat CD4 antibody (1:100, ab203034, abcam, Cambridge, MA, USA), mouse anti-rat CD8 antibody (1:100, ab33786, abcam, Cambridge, MA, USA), and rabbit anti-rat CD68 antibody (1:500, ab125212, abcam, Cambridge, MA, USA) at 4 °C overnight. Then the sections were washed and incubated with corresponding horseradish peroxidase-conjugated secondary antibodies (abcam, Cambridge, MA, USA) for 2 h at room temperature. The sections were counterstained with hematoxylin, mounted with VectaMount (Vector Laboratories, Inc., Burlingame, CA, USA), and observed under the light field of a BX51 microscope (Olympus Optical Co. Ltd., Tokyo, Japan). The pictures were taken using the cellSens Standard electronic system (Olympus Optical Co. Ltd., Tokyo, Japan). For quantification, the cornea on the sagittal section of eyeball was equally divided into 9 segments, each of which was represented by one picture. The cells positive for a particular CD staining in each picture was recorded, and the positive cell numbers in the 9 pictures were added up to represent the total number on the corneal section.

### Flow cytometry

The draining lymph nodes in each group were carefully harvested, and the lymphocytes and macrophages were analyzed using multi-parametric flow cytometry as previously described 46. In detail, the lymph nodes from PBS, rIL-10, RFP-BM-MSC and IL-10-BM-MSC groups (n = 5 / group) were harvested at day 10 post transplantation, washed twice with PBS, grinded, and filtered by cotton. The filtrates were precipitated and resuspended for the following experiments. For detecting the antigen expression on cell surface, the cell suspensions were stained with fluorochrome-conjugated antibodies to CD4 (APC-anti CD4, Biolegend, San Diego, CA, USA), CD8 (APC-anti CD8a, Biolegend, San Diego, CA, USA), and CD68 (FITC-anti CD68, Bio-Rad, Hercules, CA, USA) for 30 min at 4 °C; the cells incubated with the corresponding IgGs served as isotype controls. For Treg detection, the cells were incubated with APC-conjugated CD4 antibody as mentioned above, and then washed, spun down, resuspended, and incubated with Fixation/Permealization Buffer (Biolegend, San Diego, CA, USA) at 4 °C overnight. The next day, the cells were stained with Foxp3 (PE-anti Foxp3, Biolegend, San Diego, CA, USA) antibody according to manufacturer's instruction, the isotype control antibody to Foxp3 was included. Finally, flow cytometry was performed using FACSCalibur cytometers (BD Biosciences, San Jose, CA, USA), and data were analyzed by FlowJo software (Ashland, OR, USA).

### IL-10-BM-MSC tracking

The IL-10-BM-MSCs expressing RFP were subconjunctivally injected immediately and at day 3 after keratoplasty, and immunofluorescence was used to track the IL-10-BM-MSCs in local environment of ocular surface at day 10 post transplantation. Briefly, the frozen sections (5 μm / section) of eye balls were incubated with anti-RFP (ab152123, abcam, USA) primary antibody overnight at 4 °C following fixation, permealization, and blocking. Subsequently, the samples were incubated with Alexa 594-conjugated secondary antibody (ab150077, abcam, USA) for 1 h at room temperature, and 4',6-Diamidino-2-phenylindole (DAPI) was applied to visualize the nuclei. All sections were observed by the cellSens Standard electronic system (Olympus Optical Co. Ltd., Tokyo, Japan) under the fluorescence microscope (BX51, Olympus Optical Co. Ltd., Tokyo, Japan).

### Tissue collection and RNA sequencing

Animals were euthanized by an intraperitoneal injection of 1% pentobarbital sodium (1 ml / 100 g) at 10 d post transplantation. The grafts and the corresponding recipient beds were excised carefully using microsurgical instruments, frozen in liquid nitrogen, and stored at -80 °C (n = 12 / group). Corneas from normal rats (without transplantation and MSC treatment) were included for detecting basal levels of gene expression. Some of the collected tissues were subjected to RNA sequencing (n = 3 / group). The RNA sequencing was performed and analyzed by Novogene (Beijing, China). The rest of the tissues were used to verify the RNA sequencing results.

### Total RNA extraction, reverse transcription, and quantitative real-time PCR

Total RNA of corneas or allografts and beds (n = 9 /group) was extracted by Trizol reagent (Life Technologies, Grand Island, NY, USA) following the company's instructions. Then the RNA was quantified and reverse transcribed as described above.

The qPCR was performed in triplicate in a HT7900 Real-Time PCR System (Applied Biosystem, Foster City, CA, USA). The cDNA content of the target gene was normalized to the internal standard *β-actin* (*ActB*) gene. The reaction mixture consisted of 3 μl cDNA, 5 μl SYBR Green Master Mix (Roche, Branford, CT, USA), and the primers for lncRNA 003946 (Table 3). The pooled cDNA sample was serially diluted and used as templates to generate a standard curve between Ct values of each gene and logarithm of cDNA template concentrations. The standard curves also served as positive controls for qPCR, whereas the reactions using water as template served as negative controls. The PCR program was composed of 2 min pre-incubation at 50 °C, 10 min denaturation at 95 °C, followed by 40 cycles of 15 s at 95 °C and 1 min at 60 °C. A dissociation stage was added to check amplicon specificity. The relative target gene expression was determined using a comparative threshold cycle (2^-∆∆Ct^) method.

### Construction of lentiviral vectors containing shRNAs against lncRNA 003946

The lentiviral vectors containing three shRNA sequences against different parts of lncRNA 003946 were constructed. The sequences of the shRNAs were as following:

shRNA1: GTCTGTCCTTGAGTCAATTATCTCGAGATAATTGACTCAAGGACAGACT;

shRNA2: CTGAAGAATTGGCTGTAATTACTCGAGTAATTACAGCCAATTCTTCAG;

shRNA3: GACTAACCCTGTGACATATATCTCGAGATATATGTCACAGGGTTAGTC.

After construction, the lentiviruses carrying three shRNAs to lncRNA 003946 were packaged, concentrated, and tittered by Cyagen (Santa Clara, CA, USA).

### Selection of the most effective shRNA against lncRNA 003946

The Lewis rats were randomly allocated to 5 groups, including normal group, shRNA1 group, shRNA2 group, shRNA3 group, and scramble group, with 12 rats in each group. The titers of lentiviruses were equally adjusted to 1 × 10^10^ integration units (IU)/ml. Ten microliters of the concentrated lentivirus were subconjunctivally injected into the right eyes of the corresponding group of rats. At 5 d post injection, five eyeballs from each group were harvested, embedded in Tissue-Tek O.C.T. compound (Sakura Finetek, Torrance, CA, USA), frozen in liquid nitrogen, and sectioned into 5 μm thickness. Immunofluorescence was performed as previously described 43, 44, 47. Briefly, the sections were post-fixed in 4% PFA, washed with PBS, permealized with 0.03% sodium dodecyl sulfate, and blocked with 10% goat serum. Then the sections were incubated with a rabbit primary antibody to GFP (1:2000, ab6556, abcam, Cambridge, MA, USA) at 4 °C overnight. The next day, the sections were washed with PBS, incubated with an Alexa 488-conjugated goat anti-rabbit IgG H&L (1:1000, ab150077, abcam, Cambridge, MA, USA) for 2 h at room temperature. The sections were observed under a confocal microscope (LSM800, Zeiss, Germany) after mounting with DAPI-containing ProLong Gold Antifade reagent (Life Technologies, Grand Island, NY, USA). The representative pictures were taken by the cellSens Standard electronic system (Olympus Optical Co. Ltd., Tokyo, Japan). At 15 d after injection, the rest of rats were euthanized by an overdosed pentobarbital sodium as described above, the corneas were collected, and the expression of lncRNA 003946 was analyzed by qPCR. The shRNA3 that could downregulate the expression of lncRNA 003946 most efficiently was selected for the following experiments.

### Knockdown of lncRNA 003946 in the rats receiving penetrating keratoplasty

The rats were randomly divided into 5 groups, including PBS group, RFP-BM-MSC group, IL-10-BM-MSC group, scramble+IL-10-BM-MSC group, and shRNA3+IL-10-BM-MSC group. Each group had 16 animals. At 5 d prior to transplantation (day -5), shRNA3+IL-10-BM-MSC group of rats were subconjunctivally injected with 10 μl lentivirus carrying shRNA3; scramble+IL-10-BM-MSC group received an injection of 10 μl lentivirus carrying scramble. Other groups were subconjunctivally injected with the same volume of PBS. The penetrating keratoplasty was performed as described above. The MSC-treatments were conducted at day 0 and 3 post transplantation as described above. The grafts were monitored everyday by a slit-lamp biomicroscope, and the RI was calculated to determine the survival time of individual graft. The levels of lncRNA 003946 were determined by qPCR on Day 10 post transplantation.

### Administration of Ad-lncRNA 003946 and clinical assessments

Parts of the coding sequence of lncRNA 003946 were synthesized and spliced. The resulting full-length lncRNA 003946 coding sequence (6.1 kb) was cloned into an adenoviral expression vector downstream cytomegalovirus promoter. The adenovirus overexpressing the lncRNA (designated as Ad-lncRNA 003946) was packaged and tittered by Cyagen Biosciences Inc. (Beijing, China). The adenovirus expressing GFP (known as Ad-GFP) was also provided by the company as a control. Then the rats subjected to penetrating keratoplasty were randomly divided into PBS, Ad-GFP, and Ad-lncRNA 003946 groups (n = 8 / group). For the adenovirus injection groups, the recipient rats were subconjunctivally injected in the right eyes with the corresponding virus (1 × 10^12^ IU/ml) immediately after transplantation. The corneal grafts were monitored under a slit-lamp biomicroscope every day and the representative pictures taken as mentioned above. The RI was calculated to determine the survival time of individual graft.

### RNAscope *in situ* hybridization

At 10 d post transplantation, the comparable part of the eye balls from each group (n=6 /group) was paraffin sectioned (5 μm /section). Following the immunohistochemistry for CD68 staining as described above, the sections were pretreated and hybridized with lncRNA 003946 using the specific double Z probes designed by Advanced Cell Diagnostics (Newark, CA, USA). The probes targeting the gene encoding dihydropyridinedicarboxylic acid reductase of Bacillus subtilis (*dapB*) were included as negative control. The signaling amplification and detection were performed using the RNAscope Multiplex Fluorescent Reagent Kit (Advanced Cell Diagnostics, Newark, CA, USA) according to the manufacturer's protocol. The stained sections were observed under the light field of a BX51 microscope (Olympus Optical Co. Ltd., Tokyo, Japan). The pictures were taken using the cellSens Standard electronic system (Olympus Optical Co. Ltd., Tokyo, Japan). The ratio of the CD68^+^ cells co-expressing lncRNA 003946 over the total CD68^+^ cells was calculated in each section. The ratios were compared among all experimental groups.

### Manipulations of bone marrow-derived antigen-presenting macrophages and their effects on T-cell proliferation

Macrophages were derived from bone marrow isolated from rat hind limbs as previously described 48, 49. Briefly, the bone marrow from hind limbs of Lewis rats was flushed with PBS containing 1% Penicillin / Streptomycin (Thermo Fisher Scientific, Waltham, MA, USA). Cell suspension was cultured for 5 d in peri-dishes (Corning, Corning, NY, USA) with DMEM supplemented with 10 ng/ml Colony Stimulating Factor (ProSpec, Rehovot, Israel), 10% Fetal Bovine Serum (Thermo Fisher Scientific, Waltham, MA, USA), and 1% Penicillin/Streptomycin (Thermo Fisher Scientific, Waltham, MA, USA). Culture media were changed every other day. On day 4, the macrophages were infected by the lentivirus carrying shRNA3 or scramble and by the adenovirus carrying lncRNA 003946 or GFP with equal titers. The macrophages without virus infection were also included. The macrophages (MΦ) were then divided into 8 groups: MΦ + PBMC, MΦ + BM-MSC + PBMC, MΦ + IL-10 + PBMC, MΦ + IL-10-BM-MSC + PBMC, MΦ + lenti-shRNA + IL-10-BM-MSC + PBMC, MΦ + lenti-scramble + IL-10-BM-MSC + PBMC, MΦ + adeno-lncRNA + PBMC, and MΦ + adeno-GFP + PBMC groups (n = 6 / group). On day 5, all the macrophages were dislodged and seeded in the corresponding wells (1 × 10^6^ cells/well) of the 24-well plates (Corning, Corning, NY, USA). Then the irradiated BM-MSCs or IL-10-BM-MSCs (15 Gy, 1 × 10^5^ cells/well) or rIL-10 (100 pg / ml) were administered to the corresponding experimental groups. Twenty-four hours later, 0.1 ml PBMCs (1 × 10^6^ cells/ml) isolated from the blood of corneal allograft rejection rats were added to all the 8 groups of cells mentioned above, an extra group containing only PBMCs was included. In another 48 h, 0.1 ml supernatants containing PBMCs from each well were transferred to a new 96-well plate (Corning, Corning, NY, USA). The BrdU incorporation was examined as described above.

### Statistics

The statistical analyses were performed using Statistic Program for Social Sciences 20.0 (IBM SPSS Inc., NY, New York, USA). The Gaussian distribution and homogeneity of variance of all the data were determined by *D'Agostino-Pearson omnibus normality test* and *Levene test*, respectively. All data were expressed as Mean ± SEM. *Kaplan-Meier method* was used to compare the survival time of corneal allografts. The levels of IL-10 secreted by BM-MSCs and IL-10-BM-MSCs were compared by *2-tailed Student's t-test*. Other quantitative results were analyzed by *One-way ANOVA* followed by *Tukey or Dunnett's T3 post hoc* for pairwise comparisons. *P* value less than 0.05 was considered statistically significance.

## Supplementary Material

Supplementary figures and table.

## Figures and Tables

**Table 1:** Quantity of data output

Sample	Raw reads	Clean reads	Clean bases	Error rate (%)	Q20 (%)	Q30 (%)	GC content (%)
Normal	90282302	86025374	12.9G	0.01	97.78	94.23	47.65
PBS	86776768	82681444	12.4G	0.01	97.72	94.09	49.44
RFP-BM-MSC	85391680	81303846	12.2G	0.01	97.71	94.08	49.05
IL-10-BM-MSC	95979516	91102056	13.67G	0.01	97.73	94.13	49.82

**Table 2:** The evaluating criteria for corneal allograft rejection

Pathologies	Scoring criteria	
Opacity	0: complete transparent graft	
	1: mild graft opacity	
	2: moderate graft opacity, but iris texture visible	
	3: increased opacity, but pupil visible	
	4: complete opacity and pupil not visible	
Edema	0: no edema	
	1: moderate edema	
	2: obvious edema with graft thickening	
Vascularization	0: no vascularization	
	1: new vessels growth to 25% of graft radius	
	2: new vessels growth to 50% of graft radius	
	3: new vessels growth to 75% of graft radius	
	4: new vessels growth to center of graft	

**Table 3:** The PCR primers in this study

Primers	Purposes	Sequences
*IL-10*-F	qPCR	ATGCCTGGCTCAGCACTGCTATGT
*IL-10*-R	qPCR	TCAATTTTTCATTTTGAGTGTCAC
*lncRNA 003946*-F	qPCR	GGGAGCACGCCGCTAAG
*lncRNA 003946*-R	qPCR	ATACTTCCACATCATAATCCATCATAGAA
*β-actin*-F	qPCR	TCTGTGTGGATTGGTGGCTCTA
*β-actin*-R	qPCR	CTGCTTGCTGATCCACATCTG
*WPRE*-F	detection	CGCTGCTTTAATGCCTTTGT
*WPRE*-R	detection	GAGATCCGACTCGTCTGAGG

## Abbreviations

*ActB*: *β-actin*
Ad-lncRNA 003946: adenovirus carrying lncRNA 003946
Ad-GFP: adenovirus carrying GFP
APCs: antigen-presenting cells
BM-MSCs: bone marrow-derived mesenchymal stem cells
BrdU: bromodeoxyuridine
*dapB*: *dihydropyridinedicarboxylic acid reductase of Bacillus subtilis*
DAPI: 4',6-Diamidino-2-phenylindole
DPBS: Dulbecco's phosphate buffered saline
ELISA: enzyme-linked immunosorbent assay
GO: Gene Ontology
IL-10: Interleukin-10
IL-10-BM-MSCs: BM-MSCs overexpressing IL-10
KEGG: Kyoto Encyclopedia of Genes and Genomes
lncRNAs: Long noncoding RNAs
MΦ: macrophages
MSCs: mesenchymal stem cells
OD: optical density
PBMCs: peripheral blood mononuclear cells
PBS: phosphate-buffered saline
PFA: paraformaldehyde
qPCR: quantitative real-time PCR
recombinant rat IL-10: rIL-10
TU: transduction units
*WPRE*: *Woodchuck Hepatitis Virus Posttranscriptional Regulatory Element*
