# Supplementary material for: Lentivirus-mediated IL-10-expressing Bone Marrow Mesenchymal Stem Cells promote corneal allograft survival via upregulating lncRNA 003946 in a rat model of corneal allograft rejection
Source: Theranostics. 2020 Jul 9;10(18):8446–67. doi: 10.7150/thno.31711 (PMC7381730; doi:10.7150/thno.31711)
Supplement: Supplementary file 1 — Supplementary figures and table. [file thnov10p8446s1.pdf]

Supplementary materials

Table S1: Differentially expressed lncRNAs in allografts and their predicted target mRNAs

Transcript ID	$-\log_{10}$(p value)	Fold of changes	Regulation	Target genes
lnc_000734	3.48	23.71	up	*Nlrp1b*
lnc_004617	3.02	11.88	up	*Zcchc18; Fam199x; Esx1*
lnc_001305	2.57	24.42	up	*Fryl; Ociad1*
lnc_001529	2.29	21.15	up	*Spryd7; Trim13; Kcnrg*
lnc_003946	1.81	20.84	up	*Plxnb2; Ppp6r2; Miox; Tubgcp6; Sbf1; Hdac10; Adm2; Mapk11; Lmf2; Dennd6b; Ncaph2; Mapk12; Sbf1*
lnc_004156	1.81	15.86	up	*Slc38a4*
lnc_001202	1.46	11.34	up	-
lnc_001483	2.66	15.27	down	*Il20ra; Slc35d3; Pex7*
lnc_004094	2.23	19.30	down	*Ncoa3; Sulf2*
lnc_000463	2.08	20.44	down	*Spns1; Lat; Cd19; Rabep2; Nfatc2ip*
lnc_002935	2.04	18.89	down	-
lnc_004368	2.00	13.50	down	*Nck1; Slc35g2; Il20rb*
lnc_001594	1.82	13.35	down	*Zfp868*
lnc_000031	1.71	14.76	down	*Prr3; Gnl1*
lnc_003226	1.51	12.31	down	-
lnc_002606	1.33	14.25	down	*RT1-S2; Dhx16 RT1-T24-3 Atat1; RT1-N2; RT1-N3; Abcf1; RT1-T24-1; RGD1302996; Ppp1r10; RT1-T24-4; RT1-S3; RT1-O1; Mrps18b*

Supplementary figure 1

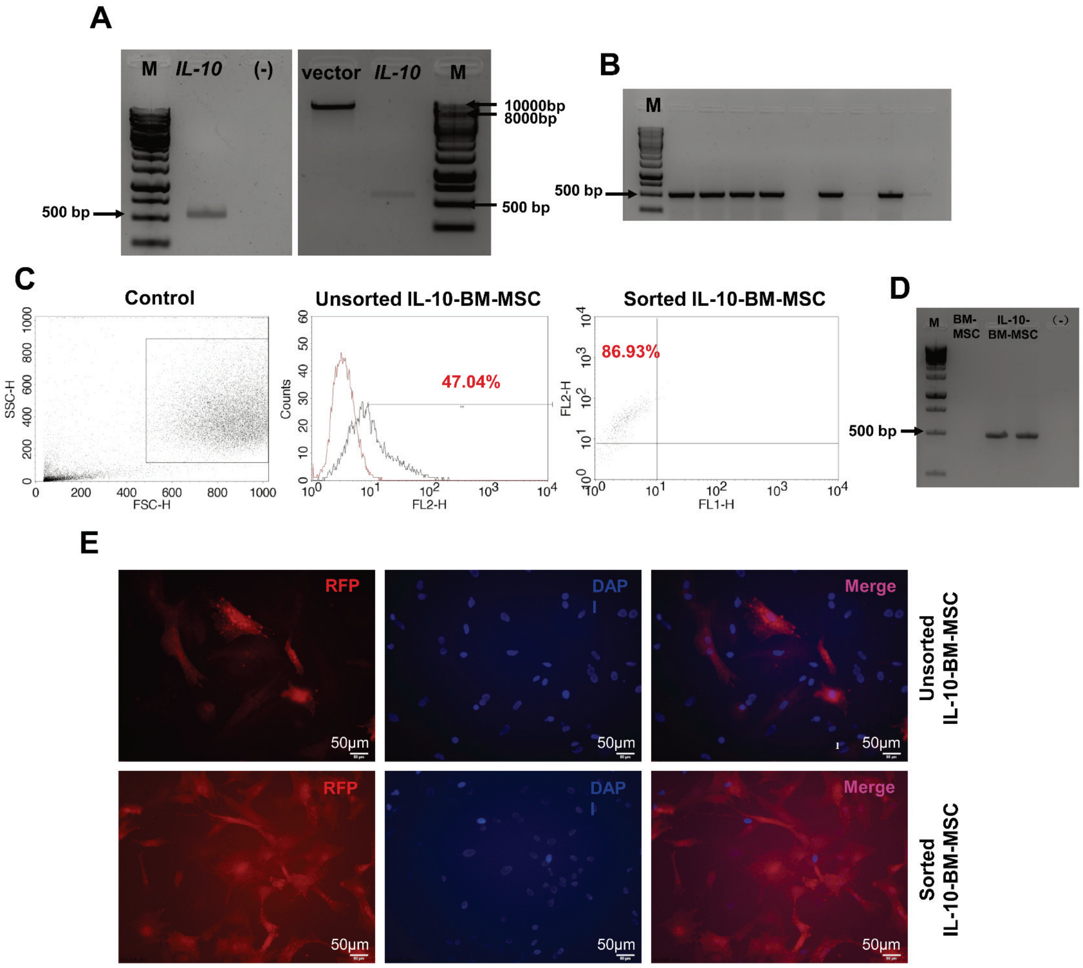

Supplementary figure 1. Generation of a pure population of BM-MSCs overexpressing IL-10. (A) The amplified rat *IL-10* cDNA fragment was visualized on an agarose gel. (-) indicates the PCR reaction using water as template. The relative concentrations of linearized lenti-vector and *IL-10* PCR product were compared. (B) The positive clones of *IL-10* cDNA-containing recombinant lentiviral vector were screened by PCR. (C) The gate of flow cytometry was set using plain BM-MSCs. The transduction efficiency of Lenti-IL10 virus was 47.04%. The purity of IL-10-BM-MSCs reached 86.93% after sorting with flow cytometry. (D) A 489 bp *WPRE* fragment that is unique on the lentiviral vector was detected in IL-10-BM-MSC's, but not BM-MSC's genome. (-) indicates the PCR reaction using water as template. (E) The representative pictures of IL-10-BM-MSCs before and after flow cytometry sorting. Scale bar = 50 μm.

Supplementary figure 2

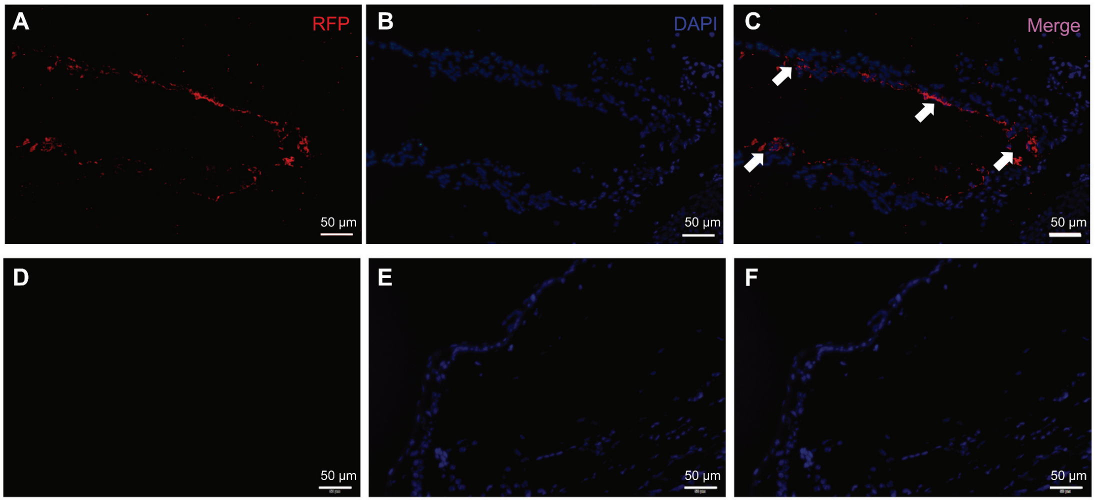

Supplementary figure 2. IL-10-BM-MSC tracking. IL-10-BM-MSCs expressing RFP were subconjunctivally injected immediately and day 3 after keratoplasty, and immunofluorescence staining pf RFP was used to track the IL-10-BM-MSCs on day 10 post transplantation. The representative images of Alexa 594-conjugated anti-RFP staining (A), DAPI nuclear staining (B), and merge (C) were shown. The staining without the anti-RFP primary antibody (D) served as a negative control, and its corresponding DAPI staining (E) and merge (F) were also shown. Scale bar = 50 µm.

Supplementary figure 3

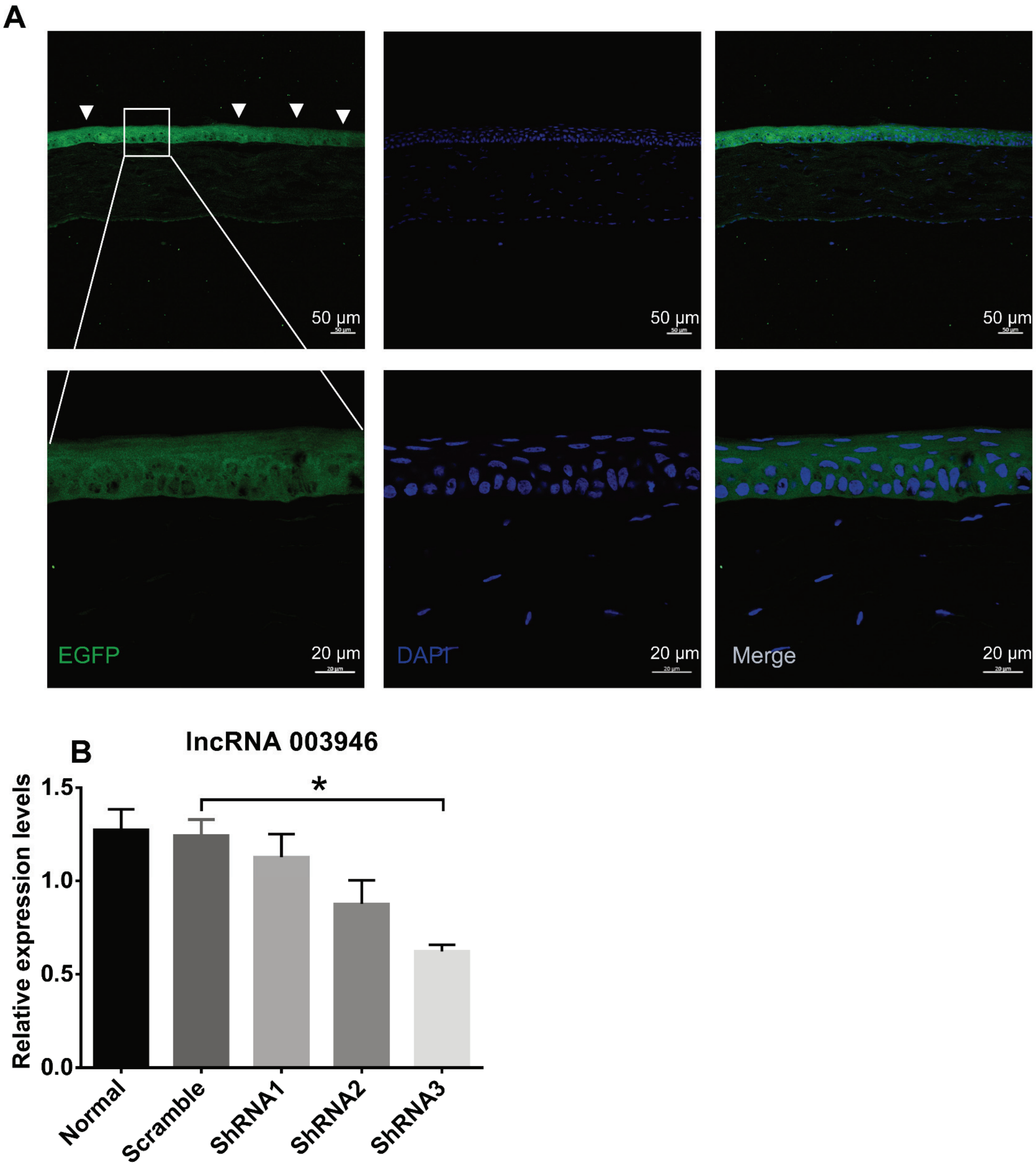

Supplementary figure 3. Selecting the most effective shRNA against lncRNA 003946 *in vivo*. (A) The lentiviruses carrying 3 shRNAs against lncRNA 003946 were subconjunctivally injected into the recipient Lewis rats. The efficiency of virus transduction and the shRNA expression were reflected by EGFP expression at 5 d post injection. The EGFP immunofluorescent staining, DAPI staining, and the merged pictures were shown (n=5 / group). Scale bar = 50 or 20 μm. (B) The relative expression levels of lncRNA 003946 in corneas were examined by qPCR at 15 d post injection (n= 7 / group). *$p < 0.05$.
